# Enhancement of Tb^III^–Cu^II^ Single‐Molecule Magnet Performance through Structural Modification

**DOI:** 10.1002/chem.201601971

**Published:** 2016-08-03

**Authors:** María José Heras Ojea, Victoria A. Milway, Gunasekaran Velmurugan, Lynne H. Thomas, Simon J. Coles, Claire Wilson, Wolfgang Wernsdorfer, Gopalan Rajaraman, Mark Murrie

**Affiliations:** ^1^ WestCHEMSchool of ChemistryUniversity of GlasgowUniversity AvenueGlasgowG12 8QQUK; ^2^ Department of ChemistryIndian Institute of Technology BombayPowaiMumbai, Maharashtra400 076India; ^3^ Department of ChemistryUniversity of BathBathBA2 7AYUK; ^4^ Department of ChemistryUniversity of SouthamptonSouthamptonSO17 1BJUK; ^5^ CNRSInst NEEL & Univ. Grenoble Alpes38000GrenobleFrance

**Keywords:** copper, heterometallic complexes, lanthanides, magnetic properties, single-molecule magnets

## Abstract

We report a series of 3d–4f complexes {Ln_2_Cu_3_(H_3_L)_2_X_*n*_} (X=OAc^−^, Ln=Gd, Tb or X=NO_3_^−^, Ln=Gd, Tb, Dy, Ho, Er) using the 2,2′‐(propane‐1,3‐diyldiimino)bis[2‐(hydroxylmethyl)propane‐1,3‐diol] (H_6_L) pro‐ligand. All complexes, except that in which Ln=Gd, show slow magnetic relaxation in zero applied dc field. A remarkable improvement of the energy barrier to reorientation of the magnetisation in the {Tb_2_Cu_3_(H_3_L)_2_X_*n*_} complexes is seen by changing the auxiliary ligands (X=OAc^−^ for NO_3_^−^). This leads to the largest reported relaxation barrier in zero applied dc field for a Tb/Cu‐based single‐molecule magnet. Ab initio CASSCF calculations performed on mononuclear Tb^III^ models are employed to understand the increase in energy barrier and the calculations suggest that the difference stems from a change in the Tb^III^ coordination environment (*C*_4*v*_ versus *C_s_*).

## Introduction

**Scheme 1:**
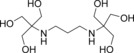
Bis–tris propane, H_6_L.

Since the discovery of the first single‐molecule magnet (SMM) the synthesis of new coordination complexes that display slow relaxation of the magnetisation and magnetic hysteresis of a purely molecular origin has been one of the main challenges in molecular magnetism.^1^, ^2^, ^3^ The great interest in SMMs is due to their potential use in technological applications, such as data storage media, quantum computing (Qubits), and spintronic devices.^4^, ^5^, ^6^ Early studies showed that the origin of the SMM behaviour arises from two main factors, the large spin ground state of the molecule (*S*) and a preferential direction for its spin (uniaxial anisotropy, *D*). A wide range of large polynuclear 3d complexes were synthesised in search of a large spin ground state.^7^, ^8^, ^9^, ^10^ However, some of these complexes—with huge *S* values—did not display the desired SMM behaviour, due to a lack of magnetic anisotropy.^9^ As magnetic anisotropy plays a critical role in the magnetic properties, lanthanide ions are good candidates for the design of SMMs. This is due to their large single‐ion anisotropy resulting from the strong spin‐orbit coupling and crystal field (CF) splitting.^11^, ^12^, ^13^ Despite the ideal qualities of lanthanide ions, some reported 4f‐based single‐molecule magnets show drawbacks, such as very efficient quantum tunnelling (QTM), which decreases the value of the energy barrier.^14^, ^15^ Therefore, the combined use of 3d/4f ions is a good strategy for the design of molecular magnets, since lanthanide ions provide the required magnetic anisotropy that is essential in SMMs and the 3d–4f exchange interaction can help to suppress QTM.^16^, ^17^ To this end, we propose the use of the ligand 2,2′‐(propane‐1,3‐diyldiimino)bis[2‐(hydroxylmethyl)propane‐1,3‐diol] (bis–tris propane, H_6_L) for the synthesis of heterometallic complexes (see Scheme 1). Bis‐tris propane is a very flexible polydentate ligand, with well‐defined coordination sites. Its skeleton has an internal {N_2_O_2_} pocket ideal for the coordination of different transition metal ions, such as Mn^II^, Fe^III^, Co^III^, Ni^II^ or Cu^II^ ions.^18^, ^19^, ^20^, ^21^ Moreover, the multiple hydroxyl arms present in H_6_L facilitate coordination with 4f metals, due to the great affinity of lanthanide ions for oxygen donors.^22^ Given the previously reported 3d,3d′ heterometallic compounds with H_6_L, the importance of Cu^II^ ions in the synthetic procedure is significant. In such complexes, the Cu^II^ atoms occupy the internal {N_2_O_2_} pocket of the ligand to the detriment of other 3d ions present in the reaction media (e.g., Mn, Zn), and thus direct and control the final molecular assembly.^20^, ^21^ In addition, Cu^II^ ions display a flexible coordination geometry and a strong ferromagnetic tendency in Cu⋅⋅⋅4f interactions for the heavier Ln ions.^23^, ^24^, ^25^, ^26^ Since the magnetic study of the first Cu/4f SMMs, several theoretical and experimental studies have investigated the factors that mainly influence the resultant magnetic properties.^11^, ^16^ Therefore, factors such as the choice of Ln^III^ ion (Dy^III^ and Tb^III^ being the most favoured), or ligands capable of promoting certain local symmetries around these 4f centres have been explored. More recent studies suggest that perturbations in the ligand environment of the Ln^III^ ions can cause changes in the CF that modify the overall magnetic behaviour of the complexes.^27^, ^28^

Herein, we describe the synthesis, structure, and magnetic properties of a new {Ln_2_Cu_3_(H_3_L)_2_X_*n*_} family of complexes based on the substitution of the lanthanide ion (Ln=Gd, Tb, Dy, Ho, Er), and the replacement of the auxiliary ligands (X=CH_3_COO^−^, NO_3_^−^). Slow magnetic relaxation is observed for all the {Ln_2_Cu_3_(H_3_L)_2_X_*n*_} complexes, except for the isotropic Gd^III^ analogues. We show that the substitution of the auxiliary ligands tunes the SMM properties of the final complexes, and that (NMe_4_)_2_[Tb_2_Cu_3_(H_3_L)_2_(NO_3_)_7_(CH_3_OH)_2_](NO_3_) is the single‐molecule magnet with the highest anisotropy barrier for Tb/Cu‐based compounds in zero applied dc field (Δ*E*/*k*_B_=36.0±0.2 K).

## Results and Discussion

The ability displayed by bis–tris propane (H_6_L) to direct the synthesis of Cu/3d heterometallic complexes presented in previous work (3d=Mn(II/III), Zn(II))^20^, ^21^ makes it a very attractive candidate for exploring the reactivity of the {Cu(H_6_L)} units with magnetically more interesting metal ions, such as rare earth elements. Therefore, several experiments involving different copper and lanthanide salts have been performed in order to study the reactivity of H_6_L in the presence of Ln^III^ ions. Moreover, the use of two different coordinating counterions, such as acetates and nitrates, is proposed as even a small alteration of the 4f environment could cause large changes in the magnetic properties of the complexes. The reaction between H_6_L, Cu(CH_3_COO)_2_⋅H_2_O and Ln(CH_3_COO)_3_⋅H_2_O (Ln=Gd^3+^, Tb^3+^) in the presence of Et_3_N allowed the synthesis of the complexes [Gd_2_Cu_3_(H_3_L)_2_(CH_3_COO)_6_]⋅THF⋅3 H_2_O (**1**) and [Tb_2_Cu_3_(H_3_L)_2_(CH_3_COO)_6_]⋅CH_3_OH⋅2 H_2_O (**2**). Violet plate‐like crystals of **1** and **2** were obtained by vapour diffusion of THF into the reaction solution in good yields. When nitrate salts are used in combination with NMe_4_OH a different series of Cu/4f complexes with formula (NMe_4_)_2_[Gd_2_Cu_3_(H_3_L)_2_(NO_3_)_8_(CH_3_CH_2_OH)_2_]⋅2 H_2_O (**3**), or (NMe_4_)_2_[Ln_2_Cu_3_(H_3_L)_2_(NO_3_)_7_(CH_3_OH)_2_](NO_3_) (**4**–**7**) (Tb^3+^, Dy^3+^, Ho^3+^, Er^3+^) were successfully synthesised. Blue block‐like crystals were obtained by slow evaporation of the reaction solution. The main structural difference between these two families of complexes ({Ln_2_Cu_3_(H_3_L)_2_(CH_3_COO)_6_} and {Ln_2_Cu_3_(H_3_L)_2_(NO_3_)_*n*_}, *n*=7 or 8) lies in the coordination environment around the metal centres, due partly to the solvent used and to the nature of the counterions present in the reaction media.

### X‐ray crystallographic analysis

Selected crystallographic experimental details for complexes **1**–**7** are shown in Table 1. Complexes crystallise in the monoclinic space group No. 14; **1** and **2** are reported in setting *P*2_1_/*n* and **3**–**7** as *P*2_1_/*c*. Complexes **1** and **2** are isostructural, as are **4**–**7**. Although all structures are in the same space group, in structures **4**–**7** the anionic unit has lower symmetry and the unit cell volume has doubled. The asymmetric unit of **1** and **2** contains a half molecule of [Ln_2_Cu_3_(H_3_L)_2_(CH_3_COO)_6_], molecules of water (one and a half molecules for **1**, one for **2**), and a half THF for **1** or a half MeOH for **2**. For the members of the {Ln_2_Cu_3_(H_3_L)_2_(NO_3_)_*n*_} family, the asymmetric unit contains one [Ln_2_Cu_3_(H_3_L)_2_(NO_3_)_7_(CH_3_OH)_2_]^−^ anion, two NMe_4_^+^ cations, and one NO_3_^−^ anion, with the exception of **3**. The asymmetric unit of **3** contains a half anion of [Gd_2_Cu_3_(H_3_L)_2_(NO_3_)_8_(CH_3_CH_2_OH)_2_]^2−^, one NMe_4_^+^ cation, and one water molecule. As the complexes are isostructural within the two 3d/4f families, the following descriptions of **1** and **4** are applicable to **2** and **5**–**7**, respectively.

**Table 1:** Crystal data and structure refinement parameters of complexes **1**–**7**. Complexes **1** and **2** are members of the {Ln_2_Cu_3_(H_3_L)_2_(CH_3_COO)_6_} family, whereas **3**–**7** are members of the {Ln_2_Cu_3_(H_3_L)_2_(NO_3_)*n*} family.^[a]^

	**1** (Gd)	**2** (Tb)	**3** (Gd)	**4** (Tb)	**5** (Dy)	**6** (Ho)	**7** (Er)
*T* [K]	100(2)	100(2)	100(2)	100(2)	100(2)	100(2)	100(2)
crystal system	monoclinic	monoclinic	monoclinic	monoclinic	monoclinic	monoclinic	monoclinic
space group	*P*2_1_/*n*	*P*2_1_/*n*	*P*2_1_/*c*	*P*2_1_/*c*	*P*2_1_/*c*	*P*2_1_/*c*	*P*2_1_/*c*
*a* [Å]	11.9478(8)	11.8081(8)	9.7807(2)	15.8081(2)	15.8020(2)	15.7834(2)	15.78770(10)
*b* [Å]	18.8874(13)	18.9453(13)	19.9923(4)	16.1451(2)	16.1620(2)	16.12290(10)	16.0957(2)
*c* [Å]	13.1236(9)	12.5980(9)	16.3177(3)	23.7104(2)	23.7208(16)	23.6867(3)	23.6668(2)
*β* [°]	108.0790(15)	106.663(2)	101.7210(10)	101.0060(10)	100.932(7)	100.7980(10)	100.8170(10)
*V* [Å^3^]	2815.3(3)	2699.9(3)	3124.21(11)	5940.14(12)	5948.2(4)	5920.93(11)	5907.21(10)
*Z*	2	2	2	4	4	4	4
*ρ*_calcd_ [mg m^−3^]	1.822	1.832	1.952	1.985	1.991	2.005	2.015
*μ* [mm]	3.525	3.833	3.211	3.520	3.649	3.815	3.986
*F*(000)	1546.0	1486.0	1846.0	3556.0	3564.0	3572.0	3580.0
refls collected	47 169	21 410	11 181	21 261	39 420	20 490	11 152
data/restraints/parameters	6441/534/366	6152/526/342	5707/5/435	10 887/61/845	13 420/10/827	10 516/12/829	10 733/34/825
GOF on *F* ^2^	1.066	1.045	1.108	1.070	1.030	1.032	1.050
final *R* indexes [*I*≥2*σ*(*I*)]	*R*_1_=0.0305 *wR*_2_=0.0795	*R*_1_=0.0663 *wR*_2_=0.1737	*R*_1_=0.0282 *wR*_2_=0.0658	*R*_1_=0.0207 *wR*_2_=0.0521	*R*_1_=0.0320 *wR*_2_=0.0696	*R*_1_=0.0206 *wR*_2_=0.0486	*R*_1_=0.0198 *wR*_2_=0.0471
final *R* indexes [all data]	*R*_1_=0.0372 *wR*_2_=0.0838	*R*_1_=0.0798 *wR*_2_=0.1845	*R*_1_=0.0348 *wR*_2_=0.0691	*R*_1_=0.0251 *wR*_2_=0.0544	*R*_1_=0.0406 *wR*_2_=0.0744	*R*_1_=0.0247 *wR*_2_=0.0502	*R*_1_=0.0236 *wR*_2_=0.0480
largest diff. peak/hole [e Å^−3^]	1.62/−0.73	6.34/−0.80	0.80/−0.54	1.22/−0.74	0.84/−0.72	0.81/−0.55	1.18/−0.63

[a] See Supporting Information for additional information related to the crystal data and structure refinement parameters.

**Figure 1:**
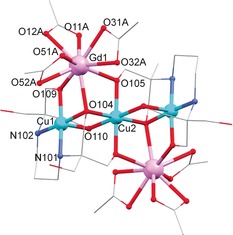
Structure of complex **1**. Hydrogen atoms and solvent molecules are omitted for clarity. Only crystallographically unique Cu, Gd, N and O atoms are labelled.

The structure of **1** contains two Gd^III^ ions coordinated to a {Cu_3_(H_3_L)_2_} linear unit through four μ‐O and two μ_3_‐O bridging atoms from two triply deprotonated H_3_L^3−^ ligands (see Figure 1). Three chelating acetate anions help to complete the coordination sphere of each lanthanide ion. The two external Cu^II^ ions of the linear unit are encapsulated by two H_3_L^3−^ ligands through O,N‐donor atoms in a [4+1] distorted environment. In order to investigate the geometry of the outer Cu^II^ ions in **1** and **2**, the parameter *τ* has been calculated.^29^ The *τ* parameter specifies the degree of distortion of the square‐pyramidal geometry in five‐coordinate structures considering their basal angles (see Figure S2 in the Supporting Information). Therefore, a *τ* value near zero is associated with a square‐pyramidal environment, whereas *τ* close to unity is related to a trigonal‐bipyramidal environment. As the *τ* parameters calculated for the external Cu^II^ ions in **1** and **2** are *τ*_Cu_(**1**)=0.21 and *τ*_Cu_(**2**)=0.22, the Cu^II^ ions are both in a distorted square‐pyramidal geometry. The central Cu^II^ ion presents a distorted octahedral geometry due to the coordination of six O(H_3_L^3−^) donor atoms, which act as bridges between the different metal centres. The two remaining hydroxyl arms on each ligand which do not bridge metal ions are uncoordinated. The symmetry analyses around the Ln^III^ ion (Ln=Gd^3+^, Tb^3+^) have been performed using the program SHAPE.^30^, ^31^, ^32^ The results of continuous shape measures (CShMs) propose a spherical capped square antiprism (*C*_4*v*_) as the closest ideal geometry for both complexes (see Table S1 of the Supporting Information). The average intramolecular Cu⋅⋅⋅M distances (M=Cu, Ln) are *d*_(Cu⋅⋅⋅Cu′)_=2.875(5) Å and *d*_(Cu⋅⋅⋅Ln)_=3.371(5) Å.

**Figure 2:**
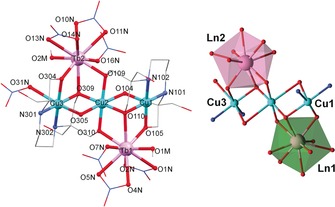
Structure of the anion (left) and detail of the metal alkoxide core (right) of **4**. Hydrogen atoms omitted for clarity. Polyhedra around Ln^III^ ions are highlighted in pink (Ln1) and light green (Ln2).

Two clear Cu‐O‐Cu′ angles could be distinguished considering the nature of the oxygen bridge, displaying average values equal to *α*_Cu‐μO‐Cu′_=68.47(1)° and *α* ${}_{\mathrm{Cu}\text{-}\mu {}_{3}O\text{-}\mathrm{Cu}{}^{'}}$ =93.30(2)°. In the same way there are two different Cu‐O‐Ln angles, with average values equal to *β*_Cu‐μO‐Ln_=104.93(5)°, and *β* ${}_{\mathrm{Cu}\text{-}\mu {}_{3}O\text{-}\mathrm{Ln}}$ =99.19(5)°. Regarding the torsion angles, there is no remarkable structural difference between them as μ‐ and μ_3_‐oxygen atoms are involved in all the angles. Considering that, the average torsion angles defined for CuOOCu′ (e.g., Cu1‐O104‐O110‐Cu2) and CuOOLn are *θ*_CuOOCu′_=168.1(1)°, and *γ*_CuOOLn_=166.8(1)°. The replacement of acetate for nitrate anions decreases the symmetry within the molecule, as there is one whole molecule in the asymmetric unit, and promotes different coordination environments around the metal ions present in the structure (see Figure 2). Consequently, the structure of (NMe_4_)_2_[Ln_2_Cu_3_(H_3_L)_2_(NO_3_)_7_(CH_3_OH)_2_](NO_3_)(**4**, Ln=Tb^3+^) contains a {Cu_3_(H_3_L)_2_} linear unit linked to two Tb^III^ ions as seen in **2**, but this time the H_3_L^3−^ ligands are coordinated to external Cu^II^ centres which have two different geometries. As shown in Figure 2, Cu1 is in a [4+1] distorted square‐based pyramidal geometry (*τ*_Cu_(**4**)=*τ*_Cu_(**5**)=0.24, *τ*_Cu_(**6**)=*τ*_Cu_(**7**)=0.25), and Cu3 is in a distorted octahedral geometry due to the coordination of an additional monodentate NO_3_^−^ ligand. The central Cu^II^ ion displays the distorted octahedral geometry seen in **1**. Two bidentate and one monodentate NO_3_^−^ ligands, plus one MeOH ligand complete the coordination environment of each nona‐coordinated Tb^III^ centre. The symmetry analyses around the Ln^III^ ion for **4**–**7** (Ln=Tb^3+^, Dy^3+^, Ho^3+^, Er^3+^) propose two different environments around the lanthanide centres, which could be related to the dissimilar coordination sphere around the neighbouring Cu^II^ atoms (see Tables S1 and S2 in the Supporting Information). The closest Ln center to the outer hexacoordinated Cu^II^ ion (Ln2, in Figure 2, right) is in a spherical capped square antiprism environment (*C*_4*v*_), whereas the one linked to the pentacoordinated Cu^II^ ion (Ln1, in Figure 2, right) displays a muffin geometry (*C_s_*). It should be noted that the coordination sphere around the metal atoms is slightly different in the case of (NMe_4_)_2_[Gd_2_Cu_3_(H_3_L)_2_(NO_3_)_8_(CH_3_CH_2_OH)_2_]⋅2 H_2_O (**3**): as both outer Cu^II^ ions are equivalent by symmetry, the three Cu^II^ centres are in a distorted octahedral geometry (Figure S1 in the Supporting Information), and an EtOH solvent molecule is coordinated to each Gd^III^ centre instead of MeOH. Shape studies performed on complex **3** reveals again structural differences with the rest of the {Ln_2_Cu_3_(H_3_L)_2_(NO_3_)_*n*_} complexes, as there is only one crystallographically unique Gd^III^ center in the low‐symmetry muffin geometry (*C_s_*). The average intramolecular distances between the different metal ions range from *d*_(Cu⋅⋅⋅Cu′)_=2.943(6)–50(6) Å, and *d*_(Cu⋅⋅⋅Ln)_=3.376(7)–20(6) Å. As a consequence of the lanthanide contraction *d*_(Cu⋅⋅⋅Ln)_ decrease along the series, *d*_(Cu⋅⋅⋅Gd)_ being the largest distance (3.420(6) Å) and consequently *d*_(Cu⋅⋅⋅Er)_ the smallest one (3.376(7) Å). Moreover, the intermolecular distances between Cu⋅⋅⋅Cu′ and Cu⋅⋅⋅Ln are slightly shorter in compounds **1** and **2** than in **3**–**7** (see Tables S3 and S4 in the Supporting Information). The different Cu‐O‐Cu′ angles show *α*_Cu‐μO‐Cu′_ values between 67.48(5)–72.18(9)°, and *α* 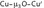 between 96.05(8)–96.80(1)°. The average Cu‐O‐Ln angles values are *β*_Cu‐μO‐Ln_=107.98(1)°, and *β* 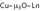
=98.55(9)°. Finally, the average torsion angles defined for CuOOCu′ and CuOOLn are *θ*_CuOOCu′_=166.3(2)° and *γ*_CuOOLn_=169.3(1)°.

A search based on the complexes reported in the Cambridge Structural Database (CSD 5.36, February 2016) reveals that there are no pentanuclear structures comparable to {Ln_2_Cu_3_(H_3_L)_2_}. Moreover, the angles between Cu^II^ ions defined as *α*_Cu‐μO‐Cu′_ for all the complexes display unusually small values (67.48(5)–72.18(9)°), with only a few examples reported in the CSD. This rare acute Cu‐O‐Cu′ angle could influence some magnetic parameters, such as the coupling between the metal ions, and therefore the overall magnetic behaviour of the complexes; this will be discussed later. Besides the rarity of the {Ln_2_Cu_3_} structure, analysis of the evolution of the magnetic properties along the 4f series make the magnetic study of these {Ln_2_Cu_3_(H_3_L)_2_} complexes interesting. Moreover, recent research points to the close relationship between the magnetic anisotropy of lanthanide ions and their local symmetry. Consequently, a comparative magneto‐structural analysis of complexes **1**–**7**, and between both {Ln_2_Cu_3_(H_3_L)_2_(CH_3_COO)_6_} and {Ln_2_Cu_3_(H_3_L)_2_(NO_3_)_*n*_} families is discussed in the following section.

### Magnetic properties

**Figure 3:**
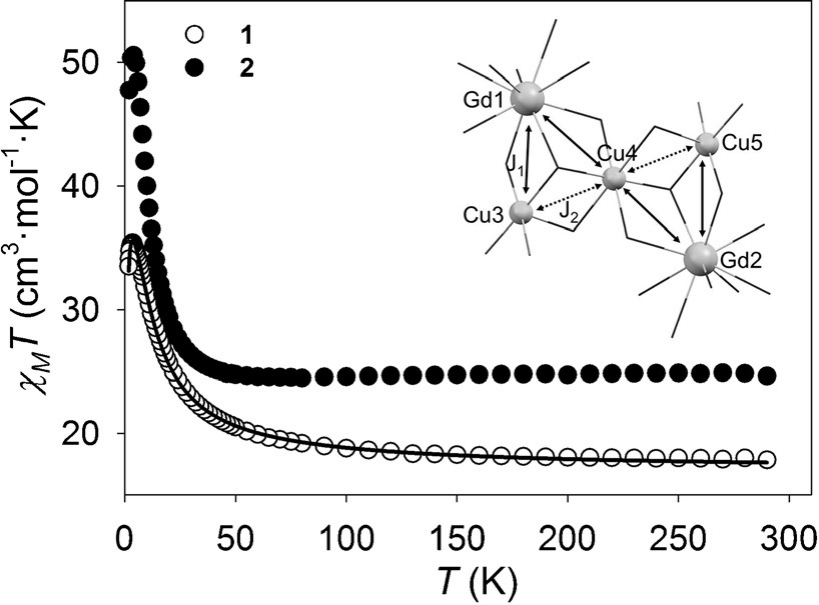
Temperature dependence of *χ*_M_ *T* for complexes **1** (Gd) and **2** (Tb) in an applied field of 1000 Oe, and magnetic model used for the fit of **1** (inset). The solid line corresponds to the fit for **1** (see text for details).

The variable‐temperature magnetic properties of complexes **1**–**2** were investigated in an applied field of 1000 Oe (Figure 3). The experimental values of *χ*_M_ *T* at 290 K for complexes **1** and **2** are consistent with those expected for three uncoupled Cu^II^ ions (*S*_Cu_=1/2, *g*_Cu_=2.11) and two Gd^III^ (^8^S_7/2_, *S*=7/2, *g*=2) or two Tb^III^ ions (^7^F_6_, *L*=3, *S*=3, *g_J_*=3/2), respectively (see Table S6 for additional information). The *g*_Cu_=2.11 value used to calculate the expected *χ*_M_ *T* value is consistent with that used in previous reported complexes presenting similar {Cu(H_6_L)} environments.^21^, ^33^ Both complexes display ferromagnetic coupling, as their experimental *χ*_M_ *T* values tend to increase with temperature, reaching maximum values of 35.43 cm^3^ mol^−1^ K at 3.4 K for **1**, and of 50.58 cm^3^ mol^−1^ K at 4.0 K for **2**.

Below these temperatures, the *χ*_M_ *T* products for **1** and **2** decrease to 33.13 cm^3^ mol^−1^ K and to 47.78 cm^3^ mol^−1^ K, respectively. The decrease in the experimental susceptibility values at low temperature could be due to a weak antiferromagnetic intermolecular interaction. As Gd^III^ is an isotropic ion, we were able to simultaneously fit the susceptibility and magnetisation data of complex **1** using the program PHI.^34^ Therefore, the fit was performed considering the magnetic model displayed in Figure 3 (right), and by applying the spin Hamiltonian shown in Equation ((1)), to give *J*_1_=1.8 cm^−1^, *J*_2_=69.7 cm^−1^ (the *g*_Cu,_ *g*_Gd_=*g* parameters were fixed at 2.11 and 2, respectively, during the fit and a small intermolecular interaction of *zJ*′=−1.5⋅10^−3^ cm^−1^ was included; *R*=99.63 %).

(1) $$H=\;-2J{}_{1}\left(\mathrm{Gd}{}_{1}\mathrm{Cu}{}_{3}+\mathrm{Gd}{}_{1}\mathrm{Cu}{}_{4}+\mathrm{Gd}{}_{2}\mathrm{Cu}{}_{4}+\mathrm{Gd}{}_{2}\mathrm{Cu}{}_{5}\right)-2J{}_{2}\left(\mathrm{Cu}{}_{3}\mathrm{Cu}{}_{4}+\mathrm{Cu}{}_{4}\mathrm{Cu}{}_{5}\right)+g{}_{\mathrm{Gd}}\mu {}_{B}\vec{B}\overset{2}{\underset{i=1}{\sum }}\vec{s}{}_{i}+g{}_{\mathrm{Cu}}\mu {}_{B}\vec{B}\overset{5}{\underset{j=3}{\sum }}\vec{s}{}_{j}$$

**Figure 4:**
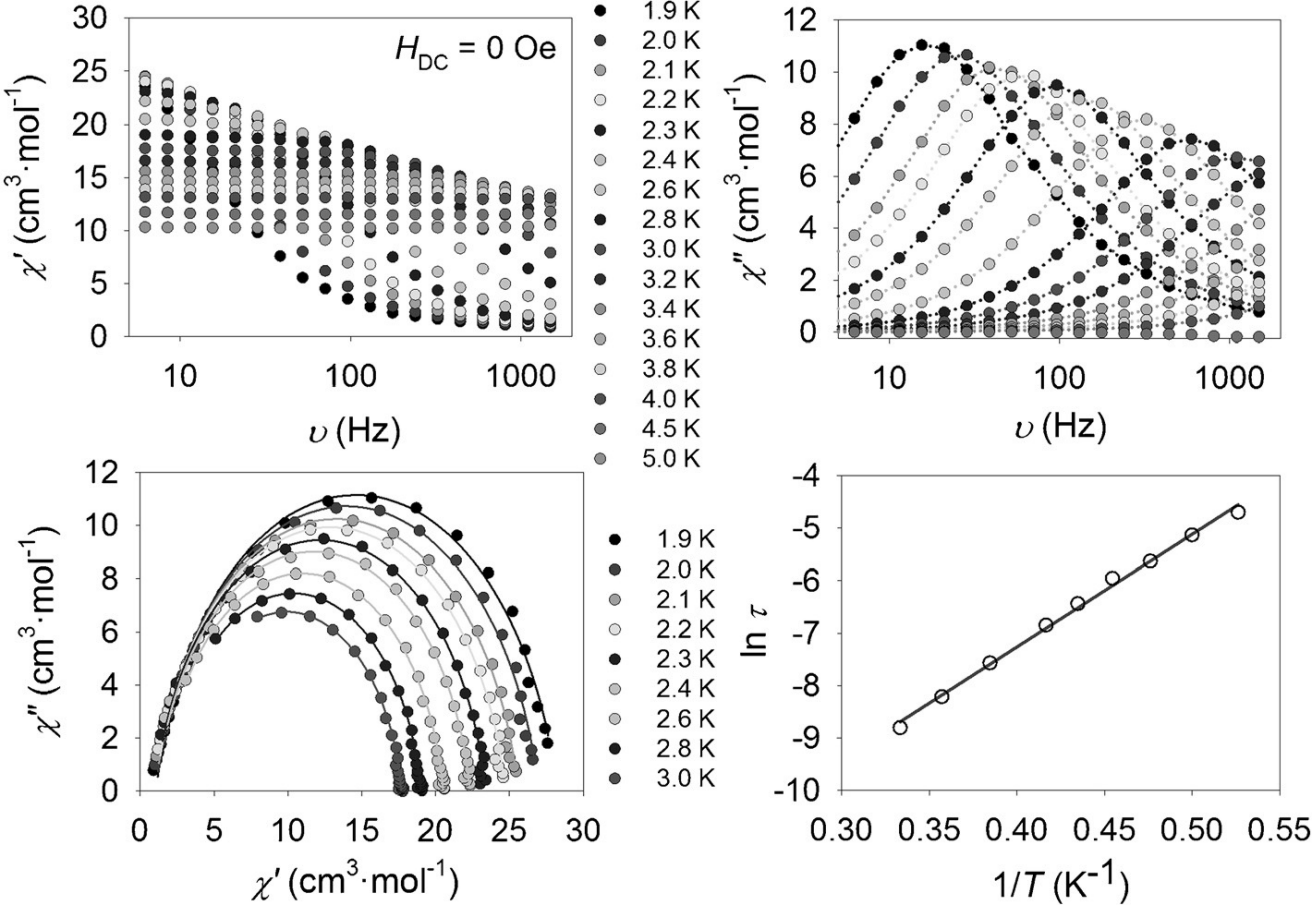
Dynamic magnetic properties for complex **2** (Tb). Top: AC magnetic susceptibility data at different frequencies in the absence of an external *H*_DC_ field. Bottom: Cole–Cole plots (left) and Arrhenius plot (right) from the AC susceptibility data. The solid lines correspond to the fit (see text for details).

The results from the fit are reasonable considering the characteristic ferromagnetic tendency of Ln^III^–Cu^II^ interactions in heteronuclear Cu/Gd complexes and that the nature and magnitude of the coupling between Cu^II^ ions (*J*_2_=69.7 cm^−1^) is consistent with the small Cu‐O‐Cu′ angles (see Table S4 in the Supporting Information). In order to study the slow relaxation of the magnetisation in complex **2**, AC susceptibility measurements as a function of the frequency over the temperature range 1.9–5 K without an applied *H*_DC_ field were performed (see Figure 4). The AC studies show slow magnetic relaxation, associated with the presence of frequency‐dependent out‐of‐phase maxima. Cole–Cole plots display a nearly symmetrical semi‐circular shape, revealing that just one single relaxation process occurs in **2** (Figure 4). In light of this, the different relaxation times (*τ*) were treated using the Arrhenius law $\tau =\tau_{0}exp\left(\Delta E/k_{B}T\right)$ , to extract the pre‐exponential factor *τ*_0_ and the energy barrier Δ*E*/*k*_B_, yielding *τ*_0_=1.3×10^−7^ s and Δ*E*/*k*_B_=21.4±0.5 K (see Figure 4). Moreover, the Cole–Cole fit displays a reasonably narrow distribution of *α* parameters in a temperature range of *T*=1.9–3 K (0.09<*α*<0.12). The estimated *τ*_0_ and Δ*E*/*k*_B_ values are comparable to those reported for other {LnCu} SMMs (see Table S5 in the Supporting Information).

**Figure 5:**
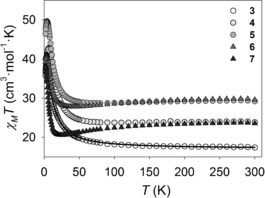
Temperature dependence of *χ*_M_ *T* for complexes **3**–**7** in an applied field of 1000 Oe. The solid line corresponds to the fit for **3** (see text for details).

Static and dynamic susceptibility measurements on complexes **3**–**7** were also performed to study whether the replacement of the auxiliary ligands has an effect on the magnetic properties of the {Ln_2_Cu_3_(H_3_L)_2_} family. The plots of *χ*_M_ *T* versus *T* of **3**–**7** in an applied field of 1000 Oe are shown in Figure 5. The experimental values of *χ*_M_ *T* at 300 K for complexes **3**–**7** are consistent with those expected for three uncoupled Cu^II^ ions (*S*_Cu_=1/2, *g*_Cu_=2.11) and two Ln^III^ ions (Gd^3+^ for **3**, Tb^3+^ for **4**, Dy^3+^ for **5**, Ho^3+^ for **6**, and Er^3+^ for **7**); see Table S6 in the Supporting Information. The experimental *χ*_M_ *T* values slightly decrease along the temperature range from 300 K to 60 K, excluding **3** (Gd) which increases. A sharp increase then takes place, until the *χ*_M_ *T* products reach maxima of 37.00 cm^3^ mol^−1^ K at 2.2 K (**3**), 42.78 cm^3^ mol^−1^ K at 6.5 K (**4**), 49.65 cm^3^ mol^−1^ K at 4.0 K (**5**), 41.16 cm^3^ mol^−1^ K at 3.4 K (**6**), and 38.33 cm^3^ mol^−1^ K (**7**) at 2.2 K. Below these temperatures the experimental *χ*_M_ *T* values drop to 36.70 cm^3^ mol^−1^ K (**3**), 35.73 cm^3^ mol^−1^ K (**4**), 46.43 cm^3^ mol^−1^ K (**5**), 39.21 cm^3^ mol^−1^ K (**6**), and 37.95 cm^3^ mol^−1^ K (**7**) at 2.0 K. Therefore, all the complexes show the ferromagnetic coupling previously displayed by **1** and **2**. As both {Gd_2_Cu_3_(H_3_L)_2_} complexes **1** and **3** display isostructural metal alkoxide cores, the fit of the dc data for **3** was performed using Equation (1), yielding, *J*_1_=1.9 cm^−1^ and *J*_2_=16.7 cm^−1^ (the *g*_Cu,_ *g*_Gd_=*g* parameters were fixed at 2.11 and 2, respectively, during the fit and a small intermolecular interaction of *zJ*′=−1.1×10^−3^ cm^−1^ was included; *R*=99.72 %). The parameters obtained from the fit are consistent with ferromagnetic exchange between both Cu⋅⋅⋅Cu′ centres, and Gd⋅⋅⋅Cu centres. The coupling constant *J*_1_ related to the interaction between Gd^III^ and Cu^II^ ions for complex **3** (1.9 cm^−1^) is quite similar to that obtained for **1** (1.8 cm^−1^). On the other hand, the *J*_2_ values corresponding to the Cu⋅⋅⋅Cu′ interaction are quite different (69.7 cm^−1^ for **1** and 16.7 cm^−1^ for **3**). An explanation for the weakening of the ferromagnetic Cu⋅⋅⋅Cu′ interaction may be related to structural differences between compounds **1** and **3** as a consequence of the replacement of CH_3_COO^−^ for NO_3_^−^ anions (Tables S3 and S4 in the Supporting Information).

**Figure 6:**
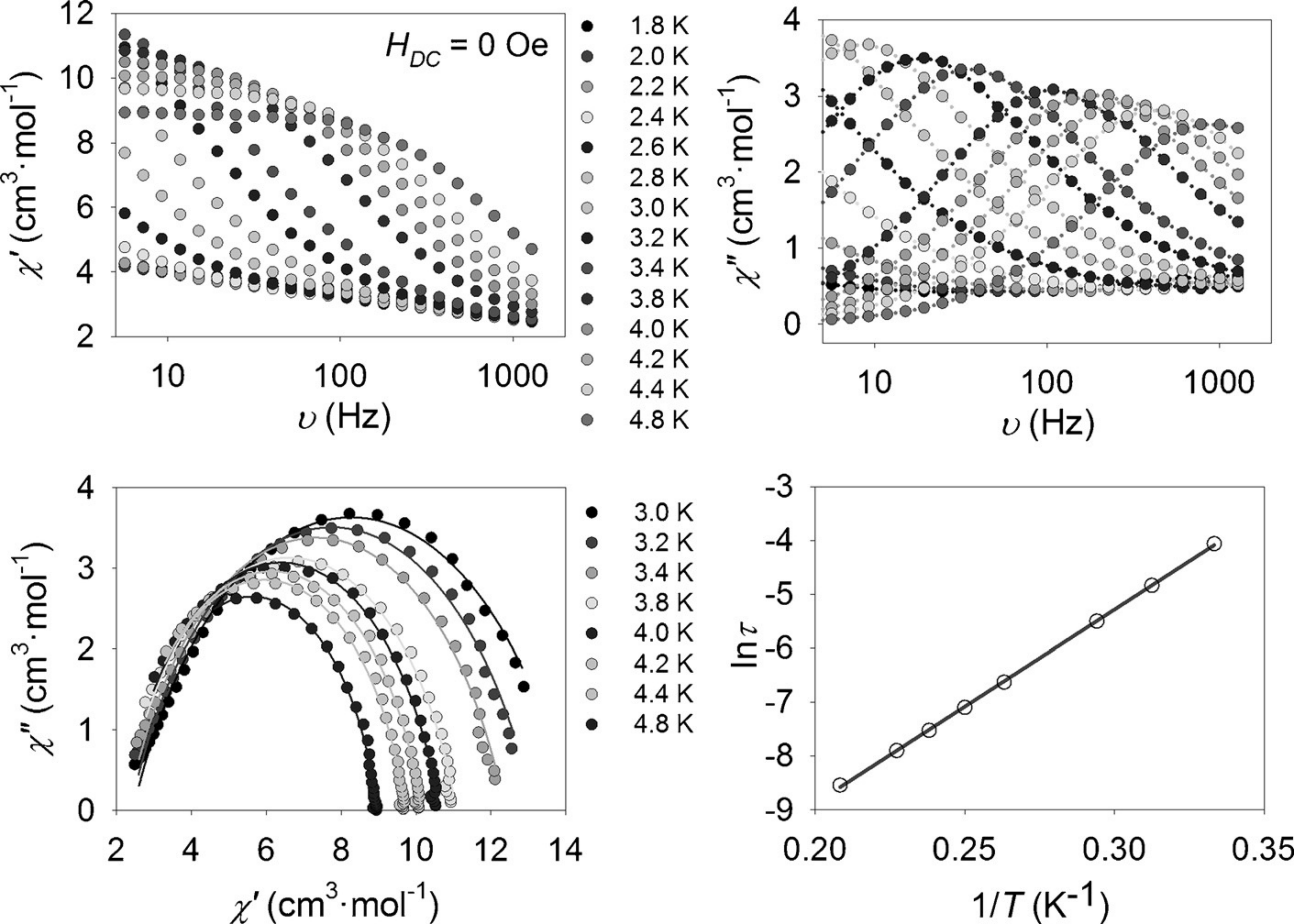
Dynamic magnetic properties for complex **4** (Tb). Top: AC magnetic susceptibility data at different frequencies in the absence of an external *H*_DC_ field. Bottom: Cole–Cole plots (left) and Arrhenius plot (right) from the AC susceptibility data. The solid lines correspond to the fit (see text for details).

The Cu^II^ centres are closer together in the case of **1**, and also the Cu‐μ_3_O‐Cu′ angles are slightly smaller in **1**. The dynamic studies for **4**–**7** (over the temperature range 1.8–8 K at *H*_DC_=0) reveal a similar behaviour for all the compounds; the appearance of a frequency‐dependent out‐of‐phase signal suggests SMM behaviour (see Figure 6 and Figures S6–S8 from Supporting Information). In the Cole–Cole plots of complex **4** it can be seen that the relaxation of the magnetisation occurs again via a single relaxation process (see Figure 6). The parameters extracted from the Arrhenius law for **4** are *τ*_0_=1.0×10^−7^ s and Δ*E*/*k*_B_=36.0±0.2 K. The substitution of the three chelating acetate ligands for two chelating nitrates, one monodentate nitrate and one MeOH ligand leads to an approximate 70 % improvement of the effective energy barrier (21.4 K for **2**, 36.0 K for **4**). It should be noted that the Δ*E*/*k*_B_ value for **4** is the largest reported value so far for Tb/Cu‐based SMMs in the absence of an applied *H*_DC_ field (see Table S5 in the Supporting Information).

**Figure 7:**
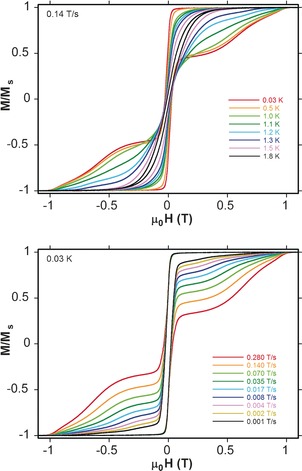
Single‐crystal magnetisation versus field hysteresis loops for complex **4**: with a constant field‐sweep rate of 0.14 T s^−1^ at different temperatures between 0.03 K and 1.8 K (top); at a constant temperature of 0.03 K with different sweep rates between 0.001 T s^−1^ and 0.280 T s^−1^ (bottom).

The enhancement of the anisotropy barrier may be attributed to changes in the electronic structure of the lanthanide ions due to changes in the local symmetry or crystal field effects related to the replacement of the auxiliary ligands.^13^, ^27^, ^28^, ^35^ To probe the SMM behaviour of **4**, single‐crystal measurements were carried out. Low‐temperature magnetisation versus field hysteresis loops are shown in Figure 7. Complex **4** shows SMM‐typical sweep‐rate‐dependent hysteresis curves with non‐zero coercivity. The coercivity displayed in the hysteresis loops decreases as the temperature rises; however, only at low enough sweep rates (below 0.001 T s^−1^) and high enough temperatures (above 1.8 K) can it be suppressed. The large step at about zero magnetic field is induced by resonant spin ground state tunnelling, which is often very strong for lanthanide compounds. At larger fields the spin relaxes through a direct relaxation process. Steps related to quantum tunnelling of the magnetisation are smeared out, as often observed for relatively large SMMs such as **4**.^36^, ^37^ Regarding the dynamic properties of **5**–**7**, the Kramers–Kronig derivate equation of the Arrhenius law ln(*χ*′′/*χ*′) = ln(*ωτ*_0_) is applied,^38^ either due to the lack of local *χ“* maxima in the AC plots or the lack of a sufficient number of maxima to fit the Cole–Cole plots, yielding the following pre‐exponential factors and energy barriers: *τ*_0_=7.5×10^−8^ s (**5**), 2.3×10^−7^ s (**6**), 1.2×10^−7^ s (**7**); and Δ*E*/*k*_B_=23.9±0.1 K (**5**), 17.2±0.2 K (**6**), 14.8±0.1 K (**7**). Again, the estimated *τ*_0_ and Δ*E*/*k*_B_ values are reasonable compared to those reported for similar {LnCu} SMMs (see Table S5 in the Supporting Information). There is a decrease of the effective barrier along the lanthanide series, with **4** showing the highest Δ*E*/*k*_B_ value, and **7** showing the lowest. This tendency is consistent with studies performed on 4f‐based single‐ion magnets that show the relationship between the atomic number of the lanthanide ions and the CF parameters (and thus SMM properties).^14^

## Theoretical studies on model complexes of 2 and 4

To understand the large barrier height observed for the Tb analogues and to probe the origin of the differences in the barrier height, we have modelled mononuclear Tb^III^ complexes derived from the X‐ray structures of complexes **2** and **4**. The two Tb^III^ ions in complex **2** are symmetry related and only one Tb^III^ ion core (model‐1) is considered. However, the Tb^III^ ions present in complex **4** are different, hence two Tb^III^ ions (model‐2 and model‐3) are considered for the calculations (see the section on Computational Details and Supporting Information Figure S9 for further information). The energy spectrum *g* tensors, relative energies and angles (*θ*) of the principal anisotropy axes of the first excited states with respect to the ground state in all three model complexes are shown in Table 2.

**Table 2:** Calculated energy spectrum, *g* tensors, relative energies and angles (*θ*) of the principal anisotropy axes of the first excited states with respect to the ground state, for ground and excited state pseudo doublets (for model‐1, model‐2 and model‐3).

	Complex **2** model‐1	Complex **4** model‐2	Complex **4** model‐3
**ground multiplet**			
*g_x_*	0	0	0
*g_y_*	0	0	0
*g_z_*	17.79	17.80	17.72
energy [cm^−1^]	0.00 and 0.45	0.00 and 0.08	0.0 and 0.32

**1st excited multiplet**			
*g_x_*	0	0	0
*g_y_*	0	0	0
*g_z_*	15.18	16.63	16.33
energy [cm^−1^]	54.03 and 56.14	58.07 and 58.47	58.93 and 63.93
angle [°]	153.62	56.48	86.29
*U*_calcd_ [cm^−1^]	54.03	58.07	58.93

**Figure 8:**
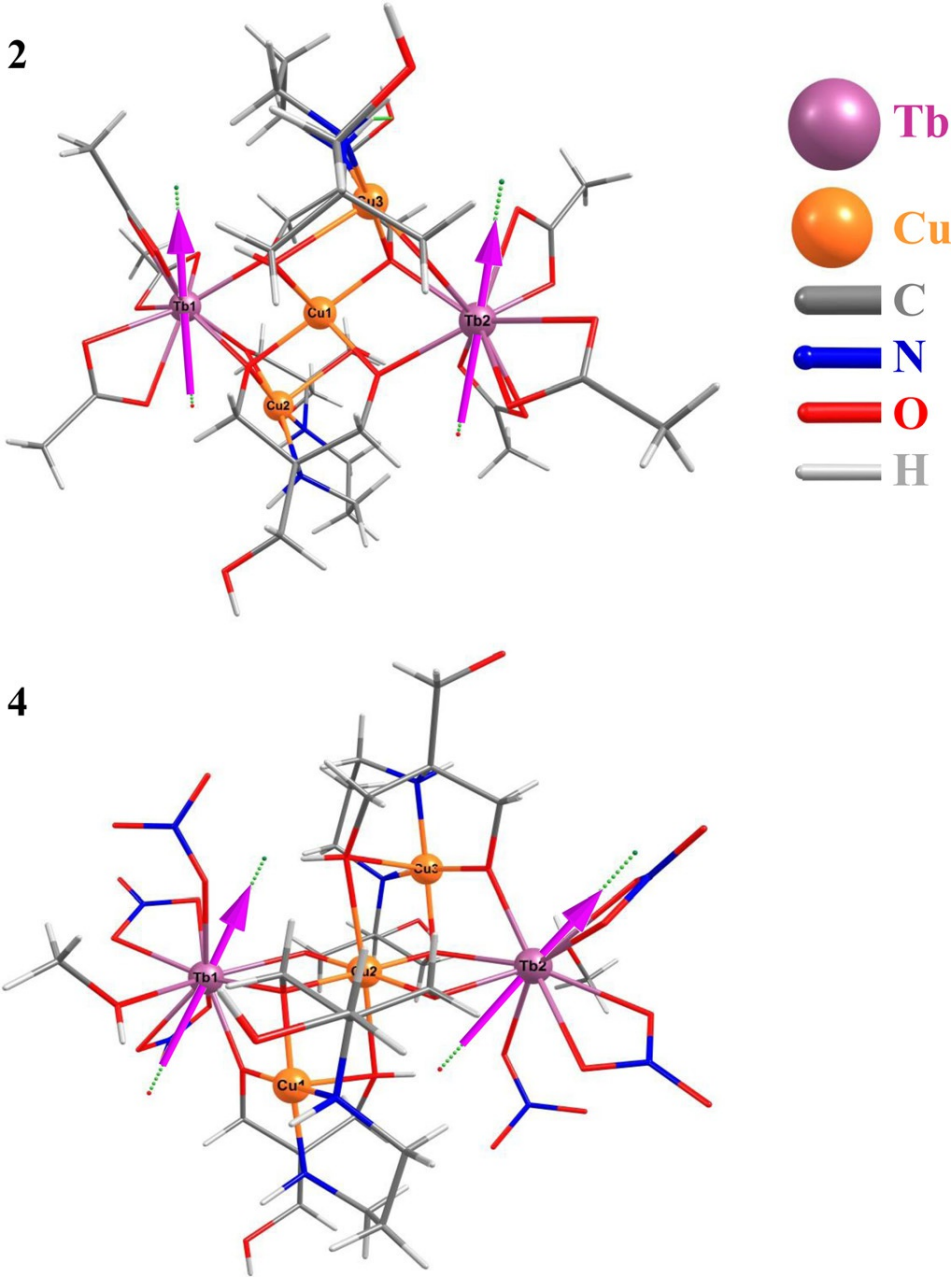
Ab initio computed orientation of *g_z_* tensors for the ground‐state Kramers doublets in complexes **2** and **4** shown with their crystal structures.

For complex **2**, as expected for the non‐Kramers ion, all the pseudo‐doublets in model‐1 are pure Ising‐type. The ground pseudo‐doublet possesses a *g_z_* of 17.79 (see ground state *g_z_* orientation in Figure 8), approaching that expected for a pure *m_J_*=± 6 state of *g_z_*≈18. A significant tunnel splitting (*Δ*_tun_) is observed within the ground multiplet (0.45 cm^−1^), suggesting that the magnetic bistability in **2** is not due to single‐ion behaviour (see Table S7 in the Supporting Information). However the presence of both Cu⋅⋅⋅Cu and Cu⋅⋅⋅Tb interactions are likely to quench the tunnel splitting as they behave like an internal applied field, leading to the observation of zero‐field SMM behaviour.^39^ If the tunneling is quenched due to this effect, the relaxation is expected to occur by means of the first excited state of Tb^III^ lying at 54 cm^−1^ (Table 2). This is due to the observation of a larger tunnel splitting for this level (Table S7 in the Supporting Information) and the *g_z_* axis being tilted significantly compared to the ground state (see Table 2). Although this value is larger than the experimental estimate (14.9 cm^−1^), our calculations do not take into account the effect of Cu⋅⋅⋅Tb exchange, intermolecular interactions and possible tunneling between states. Therefore, it represents the maximum barrier if all the above‐mentioned effects are eliminated. The orientation of the *g_z_* tensor of the ground‐state pseudo‐doublet intersects with the centre of the ligands in order to encounter the least electrostatic repulsion (see Figure 8). In contrast, for complex **4**, the tunnel splitting within the ground multiplet is small for model‐2 (0.08 cm^−1^) and significant for model‐3 (0.32 cm^−1^) (Supporting Information Tables S8 and S9). This stems from the difference in the coordination geometry in which model‐2 has a muffin‐like structure (*C_s_*) while model‐3 (and also model‐1 of complex **2**) possess a capped square anti‐prismatic geometry (*C*_4*v*_) (see Supporting Information Table S1). All the pseudo‐doublets are computed to be pure Ising‐type and the ground pseudo‐doublet for this complex possesses *g_z_* values of 17.80 and 17.72 (see ground state *g_z_* orientation in Figure 8), approaching that expected for a pure *m_J_*=± 6 state of *g_z_*≈18. The tunnel splitting in the first excited pseudo‐doublets are found to be 1.40 cm^−1^ and 5.00 cm^−1^ for model‐2 and model‐3, respectively (Supporting Information Tables S8 and S9). This gives the calculated energy barrier (*U*_cal_) 58 cm^−1^ and 59 cm^−1^, respectively, to promote relaxation via this level. Our wave function analysis reveals the Tb^III^ ground state as an admixture of 70 % |±6⟩ and small contributions from other *m_J_* levels for all the complexes. The Cu⋅⋅⋅Tb exchange interaction is expected to quench the tunneling behaviour, as shown previously leading to the observation of zero‐field SMM behavior for complexes **2** and **4**.^40^ The ground state axial ($B_{2}^{0}$ =2.02, 1.88 and 1.99 for model‐1, model‐2 and model‐3 respectively) and non‐axial ($B_{2}^{-2,-1,+1,+2}$ ) crystal field parameters are competing with each other (see Table S10 in the Supporting Information), revealing the reasons for the relatively large tunnel splitting computed. Despite this, with the internal applied field from the Cu⋅⋅⋅Tb interactions we can observe SMM behaviour due to the Ising nature of the Tb ions.

If we consider the computed parameters for model‐1 and model‐3, it is apparent that the computed behaviour is very similar between these two models, despite the fact that model‐1 has acetate ligands while model‐3 has nitrate ligands. Although the chemical environments of the ligands are different, the geometry of both models are very close to each other (see Supporting Information Table S1 for shape analysis), leading to very similar computed behaviour. This suggests that the observed difference in the magnetic behaviour between complex **2** and complex **4** stems from one of the distorted Tb^III^ ions in **4** possessing a muffin‐like coordination geometry (*C_s_*). A similar observation has been noted by us earlier for Dy^III^ SMMs.^41^, ^42^ A similar environment, if created also for the second Tb^III^ center could improve further the SMM characteristics.

## Conclusion

In conclusion, we have shown the potential of the ligand bis–tris propane to control and direct the assembly of the metal ions in the synthesis of Cu/4f complexes. The Cu^II^ ions display a preference to occupy the inner {N_2_O_2_} pocket, leaving the hydroxyl arms to bind to further oxophilic Ln^III^ ions and Cu^II^ ions. The synthesis of a new family of Cu^II^/4f heterometallic complexes with general formula {Ln_2_Cu_3_(H_3_L)_2_}, and their structural and magnetic properties were reported. All the complexes display ferromagnetic coupling in the static magnetic properties and the dynamic properties of each complex are dependent on two main factors: 1) the choice of the lanthanide ion and 2) the coordination environment of the 4f centres. Therefore, complexes with high‐magnetic anisotropy Ln^III^ ions, such as Tb^3+^ (present in **2**, **4**), Dy^3+^ (**5**), Ho^3+^ (**6**) and Er^3+^ (**7**), display the out‐of‐phase, frequency‐dependent AC signals characteristic of single‐molecule magnets. Comparing the Tb‐based complexes **2** and **4**, the AC studies show a considerable improvement (ca. 70 % increase) of the effective barrier. It should be also noted that complex **4** has the largest reported energy barrier for Tb/Cu‐based SMMs, measured in the absence of a *H*_DC_ field. Ab initio CASSCF calculations performed on the mononuclear Tb^III^ model complexes derived from complexes **2** and **4** suggest that the difference in the energy barrier arises from the structural variation around the Tb^III^ ions (*C*_4*v*_ vs. *C_s_*) and that the Cu⋅⋅⋅Tb exchange interactions help to quench the tunnelling leading to the observation of zero‐field SMM behavior.

## Experimental Section

### Materials and physical measurements

All reagents and solvents were obtained from commercial suppliers and used without further purification. The polydentate ligand H_6_L used in the synthetic routes is the commercial reagent 2,2′‐(propane‐1,3‐diyldiimino)bis[2‐(hydroxymethyl)propane‐1,3‐diol] (H_6_L).

Crystallographic data were collected for **1**–**7** at 100 K using Mo‐K*α* radiation (*λ*=0.71073 Å). For **3**, **4**, **6** and **7** a Bruker–Nonius Kappa CCD diffractometer with an Oxford Cryosystems cryostream device mounted on a sealed tube generator was used; for **1**, **2** and **5** a Rigaku AFC12 goniometer equipped with an (HG) Saturn724+ detector mounted on an FR‐E+ SuperBright rotating anode generator with HF Varimax optics (100 μm focus).^43^ All the structures were solved using SUPERFLIP^44^ and refined using full‐matrix least‐squares refinement on *F* ^2^ using SHELX2014^45^, ^46^ within OLEX2.^47^ The IR spectra were measured using a FTIR‐8400S SHIMADZU IR spectrophotometer. The microanalyses were performed by the analytical services of the School of Chemistry at the University of Glasgow. Complexes **1**–**7** show a slight hygroscopic tendency similar to that observed in previously published complexes obtained using H_6_L as a ligand. Magnetic measurements of complexes **1**–**7** were performed on polycrystalline samples constrained in eicosane, using a Quantum Design MPMS‐XL SQUID magnetometer. Data were corrected for the diamagnetic contribution of the sample holder and eicosane by measurements, and for the diamagnetism of the compounds by using Pascal's constants. Ultra‐low‐temperature (<1.8 K) hysteresis studies were performed on a single crystal sample of **4** using an array of micro‐SQUIDS (the field is oriented along the easy axis, which is found in situ by changing the field orientation with three coils).^48^

### Computational details

All ab initio calculations on the model complexes were performed with the MOLCAS 8.0 suite.^49^, ^50^, ^51^, ^52^, ^53^, ^54^, ^55^ Spin‐free wave functions were generated using the complete active space self‐consistent field (CASSCF) method. These multiconfigurational wave functions were used as input states to account for spin‐orbit coupling through the restricted active space spin state interaction‐spin orbit (RASSI‐SO) methodology.^55^, ^56^ The resulting spin‐orbit eigenstates were used for the calculation of the anisotropic magnetic properties and *g* tensors of the lowest state using a specially designed routine SINGLE_ANISO.^57^ All the atoms were represented by ANO‐RCC basis sets from the ANO‐RCC basis library included in the MOLCAS 8.0 suite. We employed the [ANO‐RCC..8s7p5d3f2g1h.] basis set for Tb^III^, and [ANO‐RCC..4s3p2d1f.] basis set for C, N and O and [ANO‐RCC..2s.] basis set for H throughout our calculations. The active space of (8,7) was used for all the models. In the configurational interaction (CI) procedure, 7 septets, 140 quintets and 195 triplets were considered. The singlet states were not included due to computational limitations. In the RASSI module, 7 septets, 105 quintets and 112 triplets were mixed by spin‐orbit coupling within the energy window of about 40 000 cm^−1^.

The two Tb^III^ ions in complex **2** are isostructural with spherical capped square antiprismatic geometry, therefore only one Tb^III^ ion core (model‐1) was considered for ab initio calculations. However, the Tb^III^ ions present in complex **4** are different, hence two Tb^III^ ions (model‐2 and model‐3) were considered for the calculations. To understand the magnetic properties of these complexes, we performed ab initio calculations of each Tb^III^ centre of the complexes. In these calculations, the effect of neighbouring Cu^II^ ions were not included and the ligands that bridge to the corresponding Cu^II^ ions were approximated. The structure of the calculated models is shown in Figure S9 in the Supporting Information.

### Synthetic methods

**[Gd_2_Cu_3_(H_3_L)_2_(CH_3_COO)_6_]⋅THF⋅3H_2_O (1)**: Et_3_N (0.13 mL, 0.9 mmol) was added to a white suspension of H_6_L (0.09 g, 0.30 mmol) in MeOH (20 mL). Cu(CH_3_COO)_2_⋅H_2_O (0.09 g, 0.45 mmol) was added, and immediately dissolved, resulting in a turquoise solution. Gd(CH_3_COO)_3_⋅H_2_O (0.11 g, 0.33 mmol) was subsequently added, turning the turquoise solution blue. The final solution was stirred and heated to 60 °C for 3 h. The initial blue solution turned violet. Violet plate‐like single crystals suitable for X‐ray diffraction were obtained by slow diffusion of tetrahydrofuran into the solution overnight. Yield 71 % (166 mg); IR: $\tilde{\nu }$ =3200, 1549, 1445, 1265, 1101, 1045, 1020, 937, 671 cm^−1^; elemental analysis calcd (%) for [Gd_2_Cu_3_(H_3_L)_2_(CH_3_COO)_6_]⋅2.25 H_2_O: C 28.00, H 4.73, N 3.84; found: C 28.29, H 4.72, N 3.56.

**[Tb_2_Cu_3_(H_3_L)_2_(CH_3_COO)_6_]⋅CH_3_OH⋅2 H_2_O (2)**: The same synthetic procedure described for **1** was followed, but using Tb(CH_3_COO)_3_⋅H_2_O instead of Gd(CH_3_COO)_3_⋅H_2_O. Violet plate‐like single crystals suitable for X‐ray diffraction were obtained by slow diffusion of tetrahydrofuran into the solution over 2 days. Yield 44 % (110 mg); IR: $\tilde{\nu }$ =3196, 1543, 1445, 1327, 1099, 1040, 1011, 934, 667 cm^−1^; elemental analysis calcd (%) for [Tb_2_Cu_3_(H_3_L)_2_(CH_3_COO)_6_]⋅CH_3_OH⋅2 H_2_O: C 28.22, H 4.87, N 3.76; found: C 28.29, H 4.72, N 3.56.

**(NMe_4_)_2_[Gd_2_Cu_3_(H_3_L)_2_(NO_3_)_8_(CH_3_CH_2_OH)_2_]⋅2 H_2_O (3)**: H_6_L (0.28 g, 1 mmol) and tetramethylammonium hydroxide pentahydrate (NMe_4_OH⋅5H_2_O) (0.38 g, 2 mmol) were combined in EtOH (40 mL), and heated to 60 °C for 20 min. Cu(NO_3_)_2_⋅3 H_2_O (0.51 g, 2 mmol) was added, giving a green suspension, which was then heated at 60 °C for 40 min. Gd(NO_3_)_3_⋅6 H_2_O (0.99 g, 2 mmol) was added and immediately dissolved, giving a dark blue solution. The resulting solution was heated for three hours, and then filtered. Blue block‐like single crystals suitable for X‐ray diffraction were obtained by slow evaporation of the filtrate over 2 weeks. Yield 7 % (74 mg); IR: $\tilde{\nu }$ =3393, 1651, 1493, 1333, 1296, 1072, 1017, 949, 679 cm^−1^; elemental analysis calcd (%) for (NMe_4_)_2_[Gd_2_Cu_3_(H_3_L)_2_(NO_3_)_8_(CH_3_CH_2_OH)_2_]⋅3.25 H_2_O: C 21.97, H 4.80, N 10.55; found: C 21.62, H 4.44, N 10.93.

**(NMe_4_)_2_[Tb_2_Cu_3_(H_3_L)_2_(NO_3_)_7_(CH_3_OH)_2_](NO_3_) (4)**: The same synthetic procedure described for **3** was followed, but using Tb(NO_3_)_3_⋅5 H_2_O instead of Gd(NO_3_)_3_⋅6 H_2_O, and MeOH instead of EtOH as solvent. Blue block‐like single crystals suitable for X‐ray diffraction were obtained by slow evaporation of the filtrate over several weeks. Yield 16 % (84 mg); IR: $\tilde{\nu }$ =3200, 1655, 1493, 1333, 1296, 1044, 1017, 949, 679 cm^−1^; elemental analysis calcd (%) for (NMe_4_)_2_[Tb_2_Cu_3_(H_3_L)_2_(NO_3_)_7_(CH_3_OH)_2_](NO_3_)⋅2.5 CH_3_OH: C 22.33, H 4.78, N 10.57; found: C 22.62, H 4.51, N 10.56.

**(NMe_4_)_2_[Dy_2_Cu_3_(H_3_L)_2_(NO_3_)_7_(CH_3_OH)_2_](NO_3_) (5)**: The same synthetic procedure described for **4** was followed, but using Dy(NO_3_)_3_⋅6 H_2_O instead of Tb(NO_3_)_3_⋅5 H_2_O. Blue block‐like single crystals suitable for X‐ray diffraction were obtained by slow evaporation of the filtrate over a few days. Yield (crystals) 5 % (60 mg); IR: $\tilde{\nu }$ =3206, 1655, 1493, 1333, 1296, 1015, 949, 814, 633 cm^−1^; elemental analysis calcd (%) for (NMe_4_)_2_[Dy_2_Cu_3_(H_3_L)_2_(NO_3_)_7_(CH_3_OH)_2_](NO_3_)⋅3.25 H_2_O: C 20.87, H 4.63, N 10.65; found: C 20.40, H 4.11, N 10.77.

**(NMe_4_)_2_[Ho_2_Cu_3_(H_3_L)_2_(NO_3_)_7_(CH_3_OH)_2_](NO_3_) (6)**: The same synthetic procedure described for **4** was followed, but using Ho(NO_3_)_3_⋅5 H_2_O instead of Tb(NO_3_)_3_⋅5 H_2_O. Blue block‐like single crystals suitable for X‐ray diffraction were obtained by slow evaporation of the filtrate over a few days. Yield (crystals) 26 % (237 mg); IR: $\tilde{\nu }$ =3242, 1649, 1474, 1385, 1310, 1074, 1007, 750, 679 cm^−1^; elemental analysis calcd (%) for (NMe_4_)_2_[Ho_2_Cu_3_(H_3_L)_2_(NO_3_)_7_(CH_3_OH)_2_](NO_3_)⋅6 H_2_O: C 20.28, H 4.79, N 10.34; found: C 19.76, H 4.26, N 10.47.

**(NMe_4_)_2_[Er_2_Cu_3_(H_3_L)_2_(NO_3_)_7_(CH_3_OH)_2_](NO_3_) (7)**: The same synthetic procedure described for **4** was followed, but using Er(NO_3_)_3_⋅5 H_2_O instead of Tb(NO_3_)_3_⋅5 H_2_O. Blue block‐like single crystals suitable for X‐ray diffraction were obtained by slow evaporation of the filtrate over a few days. Yield (crystals) 19 % (170 mg); IR: $\tilde{\nu }$ =3401, 1657, 1491, 1333, 1072, 1017, 949, 679, 602 cm^−1^; elemental analysis calcd (%) for [NMe_4_]_2_[Er_2_Cu_3_(H_3_L)_2_(NO_3_)_7_(CH_3_OH)_2_][NO_3_]⋅1.25 H_2_O: C 21.18, H 4.47, N 10.81; found: C 21.19, H 4.29, N 10.61.

The data which underpin this work are available at http://dx.doi.org/10.5525/gla.researchdata.309.

CCDC  1475631 (**1**), 1475632 (**2**), 1475633 (**3**), 1475634 (**4**), 1475635 (**5**), 1475636 (**6**) and 1475637 (**7**) contain the supplementary crystallographic data for this paper. These data can be obtained free of charge from The Cambridge Crystallographic Data Centre.

## Supporting information

As a service to our authors and readers, this journal provides supporting information supplied by the authors. Such materials are peer reviewed and may be re‐organized for online delivery, but are not copy‐edited or typeset. Technical support issues arising from supporting information (other than missing files) should be addressed to the authors.

Supplementary
