# Supplementary material for: Enhancement of TbIII–CuII Single‐Molecule Magnet Performance through Structural Modification
Source: Chemistry. 2016 Aug 3;22(36):12839–48. doi: 10.1002/chem.201601971 (PMC5008113; doi:10.1002/chem.201601971)
Supplement: Supplementary file 1 — Supplementary [file CHEM-22-12839-s001.pdf]

CHEMISTRY
A European Journal

Supporting Information

Enhancement of Tb^{III}–Cu^{II} Single-Molecule Magnet Performance through Structural Modification

María José Heras Ojea,[a] Victoria A. Milway,[a] Gunasekaran Velmurugan,[b] Lynne H. Thomas,[c] Simon J. Coles,[d] Claire Wilson,[a] Wolfgang Wernsdorfer,[e] Gopalan Rajaraman,*[b] and Mark Murrie*[a]

chem_201601971_sm_miscellaneous_information.pdf

Contents

Special details for crystal structures of complexes **2**, **4** and **6**. i

Fig S1 Structure of the anion in $(NMe_4)_2[Gd_2Cu_3(H_3L)_2(NO_3)_8(CH_3CH_2OH)_2]\cdot 2H_2O$ (**3**)............ i

Table S1 Shape measures of $\{Ln_2Cu_3\}$ relative to the ideal 9-vertex polyhedra shown of complexes **1** - **4** ii

Table S2 Shape measures of $\{Ln_2Cu_3\}$ relative to the ideal 9-vertex polyhedra shown of complexes **5** - **7** iii

Fig S2 Five-coordinate system $\{CuN_2O_3\}$ and equation for the structural parameter τ iv

Table S3 Summary of the average intramolecular distances for complexes **1** - **7**.................... iv

Table S4 Summary of the average angles (α, β), and torsion angles (θ, γ) for **1** - **7**................ v

Table S5 Summary of Cu/4*f* SMMs based on the structures reported in the CSD.................... v

Table S6. Summary of temperature dependence of the calculated ($\chi_M T_{cal}$) and experimental ($\chi_M T_{exp}$) susceptibility values for **1** – **7** vii

Fig S3 Magnetisation vs field for complexes **1** and **2** vii

Fig S4 Magnetisation vs field for complexes **3** - **6** viii

Fig S5 Magnetisation vs field for complex **7** viii

Fig S6 Dynamic magnetic studies of **5**.................... ix

Fig S7 Dynamic magnetic studies of **6**.................... x

Fig S8 Dynamic magnetic studies of **7**.................... xi

References xv

*Special details for crystal structures of complexes **2**, **4** and **6**.*

$[Tb_2Cu_3(H_3L)_2(CH_3COO)_6]\cdot CH_3OH\cdot 2H_2O$ (**2**): The crystal for complex **2** showed signs of a twin (2-fold rotation about 1 0 0) which we attempted to account for. There is approx. 18% overlap between the two twin components so a hklf4 format file corresponding to only one component was around 80% complete but did reduce the residual electron density to approx. 2 electrons/angstrom^3. An hklf5 format file with both components gave worse agreement factors and didn't improve the residual electron density. All results are for the data as processed without this twinning taken into account.

$(NMe_4)_2[Tb_2Cu_3(H_3L)_2(NO_3)_7(CH_3OH)_2](NO_3)$ (**4**): Disorder was modelled in one of the NMe_4^+ cations as a rotation about the N-C101 axis and the remaining 3 Me carbons were modelled over 2 partially occupied sites with occupancies of 0.72/0.28. Distance similarity restraints were applied to all N-C distance in the NMe_4^+ and the minor component was modelled with isotropic adps. One nitrate ion also has an oxygen atom disordered over two partially occupied sites with occupancy 0.75/0.25 and the minor component modelled with isotropic adp.

$(NMe_4)_2[Ho_2Cu_3(H_3L)_2(NO_3)_7(CH_3OH)_2](NO_3)$ (**6**): Disorder was present in one nitrate anion where one oxygen atom is modelled over two partially occupied sites with occupancies 0.85:0.15. The minor component is modelled with isotropic adps and distance restraints were applied to all N-O distances.

Fig S1 Structure of the anion in $(NMe_4)_2[Gd_2Cu_3(H_3L)_2(NO_3)_8(CH_3CH_2OH)_2]\cdot 2H_2O$ (**3**). C, grey; Cu, turquoise; N, blue; O, red; Tb, pink; hydrogen atoms omitted for clarity.

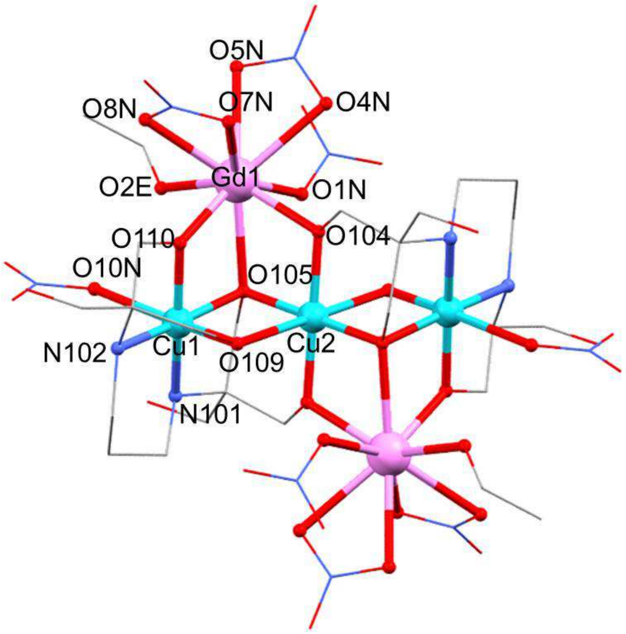

Table S1 Shape measures of {Ln_2Cu_3} relative to the ideal 9-vertex polyhedra shown of complexes [$Ln_2Cu_3(H_3L)_2(CH_3COO)_6$] (**1** - **2**) ($Gd^{3+}$, Tb^{3+}), $(NMe_4)_2[Gd_2Cu_3(H_3L)_2(NO_3)_8(CH_3CH_2OH)_2]\cdot 2H_2O$ (**3**), and $(NMe_4)_2[Tb_2Cu_3(H_3L)_2(NO_3)_7(CH_3OH)_2](NO_3)$ (**4**). The lowest $S_Q(P)$ value, and thus the closest geometry is highlighted in bold.[1]

	**$S_Q(P)$ 1**	**$S_Q(P)$ 2**	**$S_Q(P)$ 3**	**$S_Q(P)$ 4 Tb1**	**$S_Q(P)$ 4 Tb2**	**Symmetry**	**Ideal shape**
EP-9	36.757	36.630	30.410	31.370	32.088	D_{9h}	Enneagon
OPY-9	21.989	22.101	19.543	20.489	19.743	C_{8v}	Octagonal pyramid
HBPY-9	19.715	19.598	16.358	17.143	17.281	D_{7h}	Heptagonal bipyramid
JTC-9	13.828	13.772	13.601	13.135	13.524	C_{3v}	Johnson triangular cupola J3
JCCU-9	10.750	10.841	8.866	9.196	10.310	C_{4v}	Capped cube J8
CCU-9	9.463	9.677	7.920	8.490	9.534	C_{4v}	Spherical-relaxed capped cube
JCSAPR-9	2.878	2.820	3.502	2.931	2.944	C_{4v}	Capped square antiprism J10
CSAPR-9	**1.889**	**1.937**	2.608	2.144	**2.043**	C_{4v}	Spherical capped square antiprism
JTCTPR-9	3.469	3.310	2.457	2.333	2.363	D_{3h}	Tricapped trigonal prism J51
TCTPR-9	2.195	2.171	2.649	2.609	2.189	D_{3h}	Spherical tricapped trigonal prism
JTDIC-9	11.492	11.689	12.607	14.190	13.421	C_{3v}	Tridiminished icosahedron J63
HH-9	12.344	12.360	9.034	9.245	10.373	C_{2v}	Hula-hoop
MFF-9	2.252	2.377	**2.271**	**1.766**	2.174	C_s	Muffin

Table S2 Shape measures of $\{Ln_2Cu_3\}$ relative to the ideal 9-vertex polyhedra shown of complexes $(NMe_4)_2[Tb_2Cu_3(H_3L)_2(NO_3)_7(CH_3OH)_2](NO_3)$ (**5** - **7**) (Dy^{3+}, Ho^{3+}, Er^{3+}). The lowest $S_Q(P)$ value, and thus the closest geometry is highlighted in bold.[1]

	$S_Q(P)$ **5 Dy1**	$S_Q(P)$ **5 Dy2**	$S_Q(P)$ **6 Ho1**	$S_Q(P)$ **6 Ho2**	$S_Q(P)$ **7 Er1**	$S_Q(P)$ **7 Er2**	**Symmetry**	**Ideal shape**
EP-9	31.389	31.976	31.381	31.956	31.362	31.953	D_{9h}	Enneagon
OPY-9	20.633	19.859	20.764	19.914	20.851	19.952	C_{8v}	Octagonal pyramid
HBPY-9	17.253	17.391	17.289	17.383	17.400	17.444	D_{7h}	Heptagonal bipyramid
JTC-9	13.256	13.462	13.284	13.443	13.270	13.450	C_{3v}	Johnson triangular cupola J3
JCCU-9	9.206	10.332	9.050	10.232	9.038	10.258	C_{4v}	Capped cube J8
CCU-9	8.540	9.584	8.458	9.515	8.515	9.558	C_{4v}	Spherical-relaxed capped cube
JCSAPR-9	2.886	2.937	2.895	2.919	2.871	2.868	C_{4v}	Capped square antiprism J10
CSAPR-9	2.106	**2.037**	2.106	**2.025**	2.101	**1.984**	C_{4v}	Spherical capped square antiprism
JTCTPR-9	2.281	2.284	2.246	2.237	2.191	2.192	D_{3h}	Tricapped trigonal prism J51
TCTPR-9	2.586	2.168	2.587	2.146	2.603	2.122	D_{3h}	Spherical tricapped trigonal prism
JTDIC-9	14.131	13.438	14.127	13.482	14.095	13.528	C_{3v}	Tridiminished icosahedron J63
HH-9	9.325	10.443	9.305	10.404	9.346	10.436	C_{2v}	Hula-hoop
MFF-9	**1.740**	2.165	**1.733**	2.160	**1.733**	2.127	C_s	Muffin

Fig S2 Five-coordinate system {CuN_2O_3} and equation for the structural parameter τ. The α angle (corresponding to the smaller basal plane angle) is shown in red, whereas the β angle (corresponding to the bigger one) is displayed in blue. Cu, turquoise; N, blue; O, red.

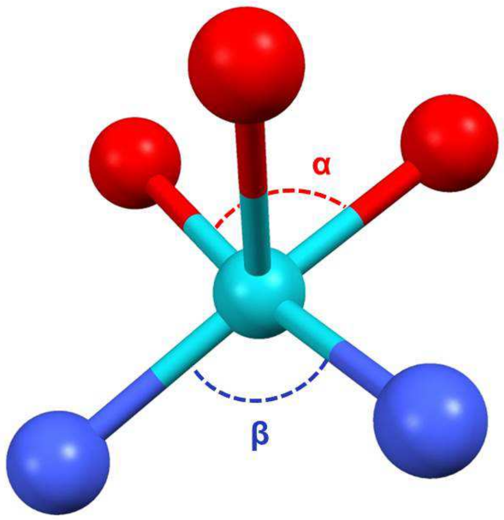

$$\tau = \frac{(\beta - \alpha)}{60}$$

Table S3 Summary of the average intramolecular distances between metal ions for complexes **1** - **7**.

	$d_{(Cu \cdots Cu')}$/Å	$d_{(Cu \cdots Ln)}$/Å
**1**	2.878(3)	3.378(5)
**2**	2.871(9)	3.364(9)
**3**	2.950(6)	3.420(6)
**4**	2.943(6)	3.409(7)
**5**	2.944(6)	3.400(7)
**6**	2.940(6)	3.390(7)
**7**	2.944(6)	3.376(7)

Table S4 Summary of the average angles (α, β), and torsion angles (θ, γ) defined by the different metal ions for complexes **1** - **7**.

	$\alpha_{Cu\text{-}\mu O\text{-}Cu'}$/°	$\alpha_{Cu\text{-}\mu 3O\text{-}Cu'}$/°	$\beta_{Cu\text{-}\mu O\text{-}Ln}$/°	$\beta_{Cu\text{-}\mu 3O\text{-}Ln}$/°	$\theta_{CuOOCu'}$/°	γ_{CuOOLn}/°
**1**	68.43(1)	93.60(4)	104.90(9)	99.47(9)	167.9(1)	166.8(1)
**2**	68.50(2)	93.00(2)	104.95(2)	98.90(2)	168.3(1)	166.7(1)
**3**	67.48(5)	96.80(1)	108.35(1)	98.90(9)	163.6(1)	170.0(1)
**4**	72.01(5)	96.05(8)	107.95(8)	98.42(7)	166.5(1)	169.0(9)
**5**	72.01(7)	96.15(1)	107.95(1)	98.35(9)	167.6(1)	169.0(1)
**6**	72.06(5)	96.24(7)	107.88(8)	98.52(7)	167.1(1)	169.2(8)
**7**	72.18(9)	96.30(1)	107.76(1)	98.50(1)	167.1(2)	169.2(1)

Table S5 Summary of Cu/4*f* SMMs based on the structures reported in the Cambridge Structural Database (CSD 5.36 version, February 2016). Energy barrier, pre-exponential factor, and magnetic DC field are indicated as $\Delta E/k_B$, τ_0, and H_{DC} respectively.

	Complex	$\Delta E/k_B$ / K (applied H_{DC}/Oe)	τ_0/s	Ref
1	$[L^4CuTb(NO_3)_2]_2(CH_3OH)_2$	4.2 (0)	$1\cdot10^{-5}$	[2]
2	$[Dy^{III}{}_3Cu^{II}{}_6L_6(\mu_3\text{-}OH)_6(H_2O)_{10}]Cl_2\cdot ClO_4\cdot 3.5H_2O$	25 (0)	$1.5\cdot10^{-7}$	[3]
3	$[LCu(O_2COMe)Tb(thd)_2]$	13.8 (1000)	$3\cdot10^{-7}$	[4]
4	$[\{Dy(hfac)_3\}_2\{Cu(dpk)_2\}]$	47 (0)	$1.1\cdot10^{-7}$	[5]
5	$[\{Cu^{II}LTb^{III}(o\text{-}van)(CH_3COO)(MeOH)\}_2]\cdot 2H_2O$	20.4 (1000)	$7.1\cdot10^{-8}$	[6]
6	$[LCuDy(hfa)_2(dmf)_2]_2$	14.7 (0)	$1.7\cdot10^{-7}$	[7]
7	$C_{24}H_{32}N_5O_{15}CuTb$	16.6 (0)	-	[8]
8	$[\{TbCu(L4)(L5)(NO_3)_2\}_2]$	17 (0)	-	[9]
9	$[TbCu(L3)(NO_3)_3(H_2O)]$	29 (1000)	-	[10]
10	$[TbCu(L3)(o\text{-}vanilate)(NO_3)(MeOH)]NO_3$	32.2 (1000)	-	[10]
11	$[H_3O][Cu_{24}Dy_8(Ph_3C\text{–}PO_3)_6(Ph_3C\text{–}PO_3H)_6(MeCO_2)_{12}(MeCO_2H)_6(OH)_{42}(NO_3)(OH_2)_6]$	4.6 (0)	$2.1\cdot10^{-8}$	[11]
12	$[TbCu(sal)(NO_3)_2(L)(MeOH)]$	32.9 (1000)	$3.0\cdot10^{-8}$	[12]
13	$[DyCu(sal)(NO_3)_2(L)(MeOH)]$	26.0 (1000)	$1.02\cdot10^{-5}$	[12]

	Complex	$\Delta E/k_B$ / K (applied H_{DC}/Oe)	τ_0/s	Ref
14	$[Cu^{II}_5Tb^{III}_4O_2(teaH)_4\{O_2CC(CH_3)_3\}_2(NO_3)_4(OMe)_4]\cdot 2MeOH\cdot 2Et_2O$	7 (0)	$1.3\cdot 10^{-5}$	[13]
15	$[Cu^{II}_5Dy^{III}_4O_2(teaH)_4\{O_2CC(CH_3)_3\}_2(NO_3)_4(OMe)_4]\cdot 2MeOH\cdot 2Et_2O$	11.9 (0)	$9\cdot 10^{-6}$	[13]
16	$[Cu^{II}_5Ho^{III}_4O_2(teaH)_4\{O_2CC(CH_3)_3\}_2(NO_3)_4(OMe)_4]\cdot 2MeOH\cdot 2Et_2O$	10 (0)	$3\cdot 10^{-6}$	[13]
17	$[Cu_2Tb_2(L)_2(dae\text{-}o)_2(NO_3)_2]\cdot 2(n\text{-}BuOH)$	14.91 (0)	$2.39\cdot 10^{-7}$	[14]
		23.76 (1000)	$7\cdot 10^{-8}$	
18	$\{[CuTb(L)(n\text{-}BuOH)_{0.5}]_2(daec)_3\}\cdot 5(DMF)\cdot 4(n\text{-}BuOH)\cdot 2(H_2O)$	12.13 (0)	$3.03\cdot 10^{-6}$	[14]
		22.04 (1000)	$2.11\cdot 10^{-7}$	
19	$[TbCu_4(L)_2(\mu_3\text{-}OH)_4(H_2O)_8(NO_3)](ClO_4)_2\cdot 6H_2O$	25.0 (3000)	$8.1\cdot 10^{-8}$	[15]
20	$[SmCu_4(L)_2(\mu_3\text{-}OH)_4(H_2O)_8(NO_3)](ClO_4)_2\cdot 6H_2O$	14.1 (3000)	$4.7\cdot 10^{-7}$	[15]
21	$[Cu(L)(C_3H_6O)Tb(NO_3)_3]$	42.3 (1000)	$7.1\cdot 10^{-10}$	[16]
22	$[Cu(L)(C_3H_6O)Dy(NO_3)_3]$	11.5 (1000)	$4\cdot 10^{-10}$	[16]
23	$[Dy^{III}_2Cu^{II}_7(OH)_2(L^2)_2(L^3)_2(OAc)_8(NO_3)_2(H_2O)_4](NO_3)_2\cdot 8.5H_2O$	18.0 (0)	$5.61\cdot 10^{-8}$	[17]
24	$[Dy^{III}_9Cu^{II}_8(NO_3)_2(OH)_{10}(L^3)_4(OAc)_{18}(H_2O)_4](NO_3)_2(OH)_3\cdot 16H_2O\cdot 10MeOH$	11.5 (0)	$1.86\cdot 10^{-6}$	[18]
25	$[Dy_2Cu_8(\mu_2\text{-}OH)_2(\mu_3\text{-}OH)_2(ClO_4)_2(HTMHSA)_4(H_2O)10]\cdot 15H_2O$	0.82 (0)	$8.02\cdot 10^{-6}$	[19]
26	$[Cu_5Dy_2(L)_2(\mu_3\text{-}OH)_4(\mu\text{-}OH_2)_2(\mu\text{-}OAc)_2(OAc)_2(OH_2)_2](NO_3)_2(H_2O)_2$	4 (0)	$3\cdot 10^{-6}$	[20]
27	$[Cu_5Ho_2(L)_2(\mu_3\text{-}OH)_4(\mu\text{-}OH_2)_2(\mu\text{-}OAc)_2(OAc)_2(OH_2)_2](NO_3)_2(H_2O)_2$	6 (900)	$3\cdot 10^{-6}$	[20]
28	$[Cu_2(valpn)_2Tb_2(N_3)_6]\cdot 2CH_3OH$	30.1 (0)	$1.1\cdot 10^{-6}$	[21]
29	$[Cu^{II}_4Dy^{III}_4(vanox)_6(Hvanox)_2(NO_3)_4(\mu\text{-}HOMe)_2]\cdot 6MeOH$	41.6 (0)	$2.1\cdot 10^{-5}$	[22]
30	$[(CuL)_2Tb(H_2O)(NO_3)_3]\cdot MeOH\cdot H_2O$	20.3 (1000)	$1.5\cdot 10^{-7}$	[23]
31	$[\{(CuL)_2Tb(H_2O)(NO_3)_3\}2bpy]\cdot 2MeOH\cdot 4H_2O$	18.0 (1000)	$1.2\cdot 10^{-8}$	[23]
32	$[Cu_6Dy_2(L^{3-})_4(NO_3)_3(OAc)(CH_3OH)_6]\cdot NO_3\cdot OAc\cdot 3CH_3OH\cdot 2H_2O$	5.2 (0)	$6.5\cdot 10^{-6}$	[24]
33	$[Cu_6Tb_2(L^{3-})_4(NO_3)_3(OAc)_2(CH_3OH)_5]\cdot NO_3\cdot CH_3OH\cdot 6H_2O$	15.6 (0)	$6.9\cdot 10^{-7}$	[24]

Table S6. Summary of temperature dependence of the calculated ($\chi_M T_{cal}$) and experimental ($\chi_M T_{exp}$) susceptibility values for **1** – **7**. *L*, *S*, g_J and ground spin term symbol are related to each lanthanide ion.

Complex	*L*	*S*	g_J	GS term symbol	$\chi_M T_{calc}$({Ln_2Cu_3}) ($cm^3 \cdot mol^{-1} \cdot K$)	$\chi_M T_{exp}$({Ln_2Cu_3}) ($cm^3 \cdot mol^{-1} \cdot K$)
**1**	0	7/2	2	$^8S_{7/2}$	17.00	17.84
**2**	3	3	3/2	$^7F_6$	24.89	24.62
**3**	0	7/2	2	$^8S_{7/2}$	17.00	17.45
**4**	3	3	3/2	$^7F_6$	24.89	23.83
**5**	5	5/2	4/3	$^6H_{15/2}$	29.59	29.19
**6**	6	2	5/4	$^5I_8$	29.39	29.57
**7**	6	3/2	6/5	$^4I_{15/2}$	24.21	23.54

Fig S3 Magnetisation vs field at different temperatures for complexes **1** (T = 2 K, 5 K) and **2** (2 K, 4 K, 6 K). The red lines correspond to the fit of **1** (see main article for details).

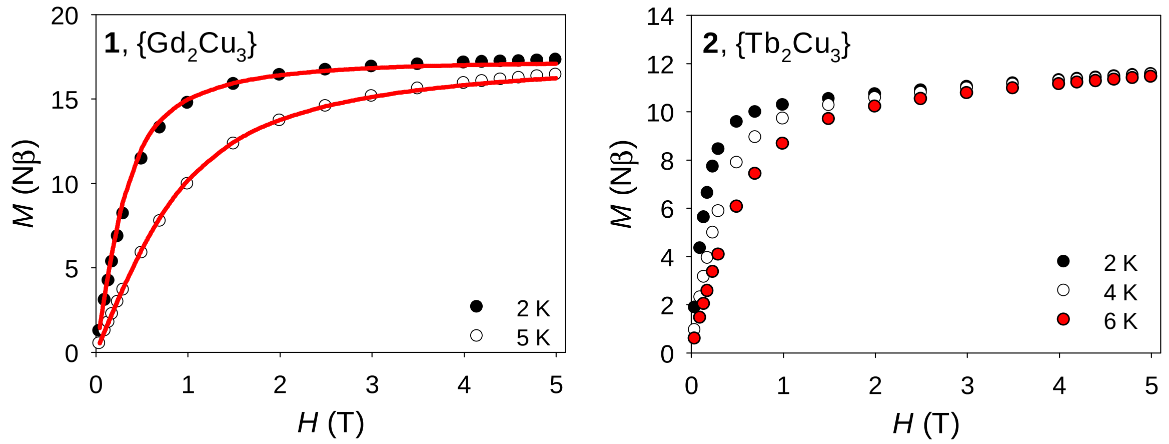

Fig S4 Magnetisation vs field at T = 2 K (black), 5 K (white) for complexes **3** - **6**.

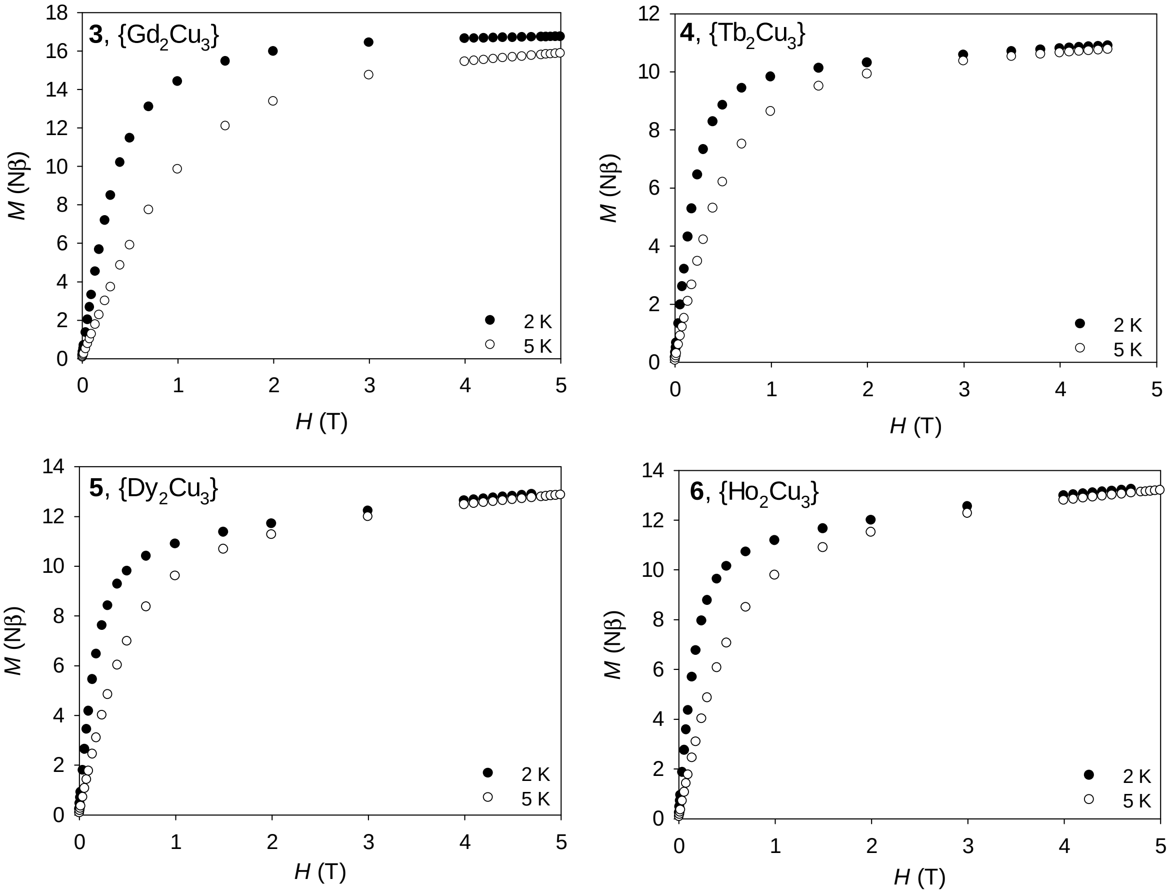

Fig S5 Magnetisation vs field at T = 2 K (black), 5 K (white) for complex **7**.

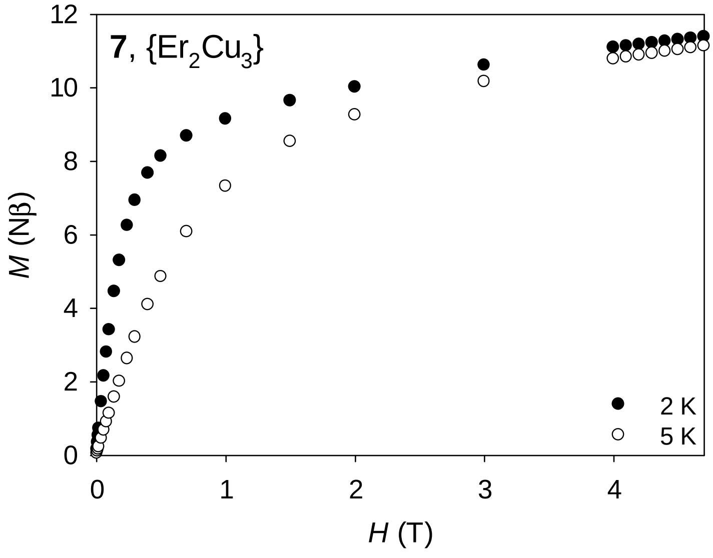

Fig S6 AC magnetic susceptibility of **5** in the absence of an external H_{DC} field (top), and natural logarithm of χ'/χ'' vs 1/T at selected frequencies of υ = 1267, 977, 476, 225, 130, 28, 10, 5, 1 Hz (bottom). Solid lines in χ'/χ'' vs 1/T correspond to fits of the data.[25]

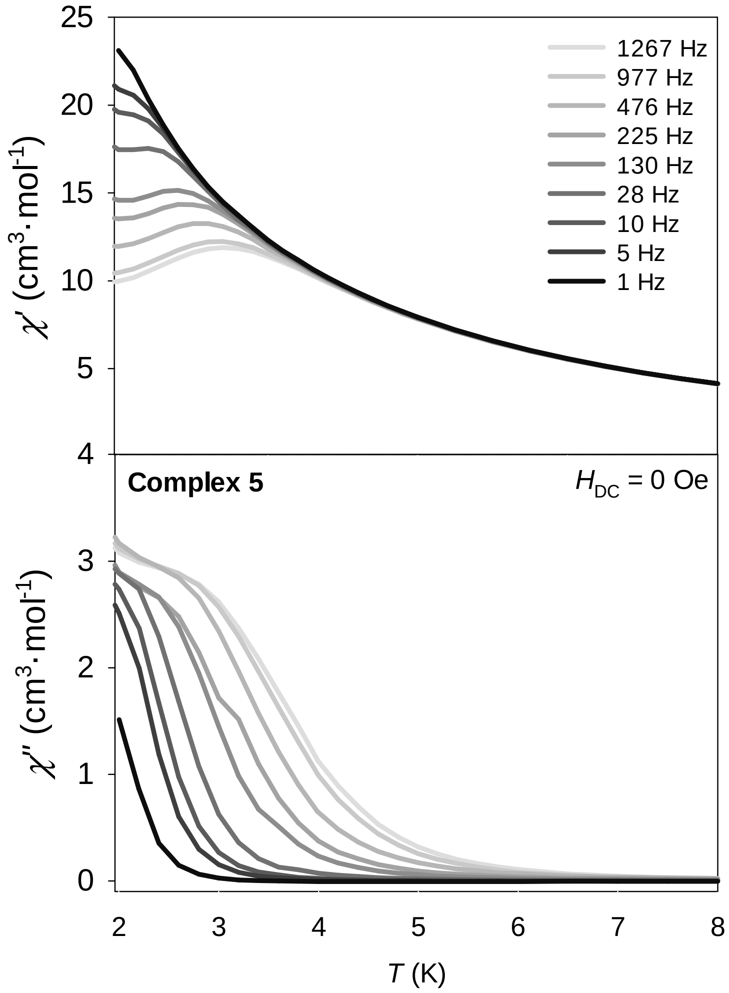

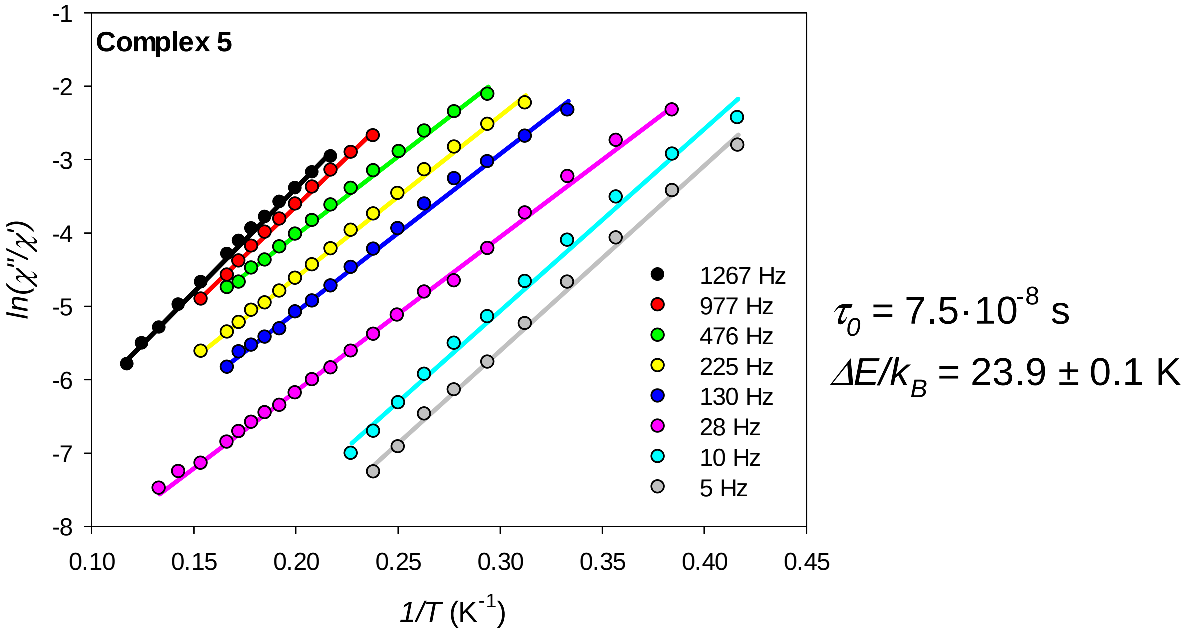

Fig S7 AC magnetic susceptibility of **6** in the absence of an external H_{DC} field (top), and natural logarithm of χ'/χ'' vs 1/*T* at selected frequencies of υ = 1267, 977, 476, 225, 130, 28, 10, 5, 1 Hz (bottom). Solid lines in χ'/χ'' vs 1/*T* correspond to fits of the data.[25]

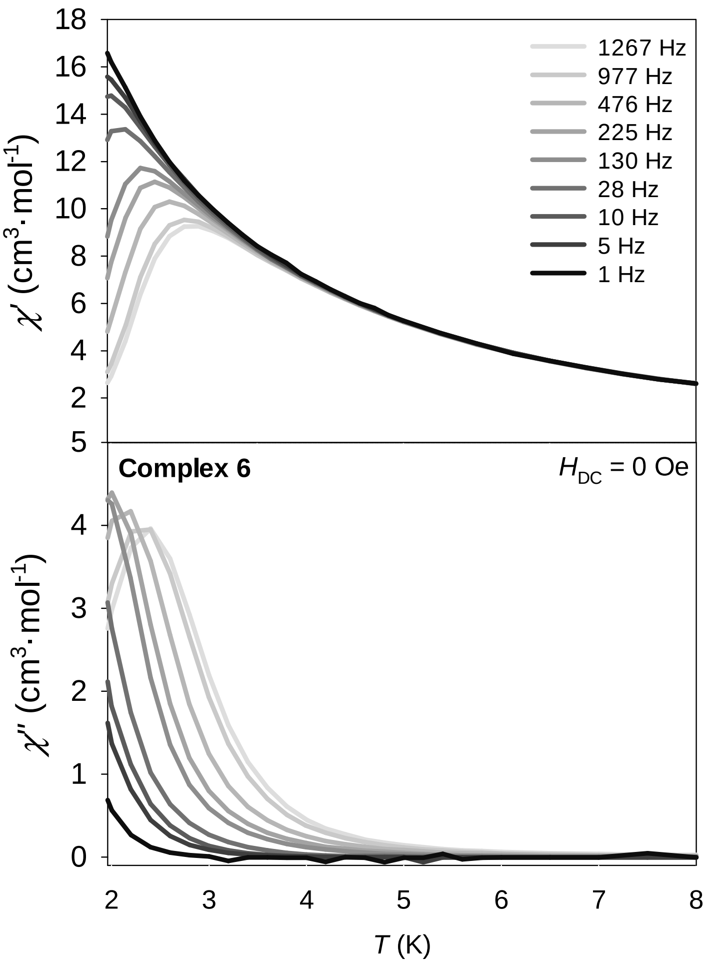

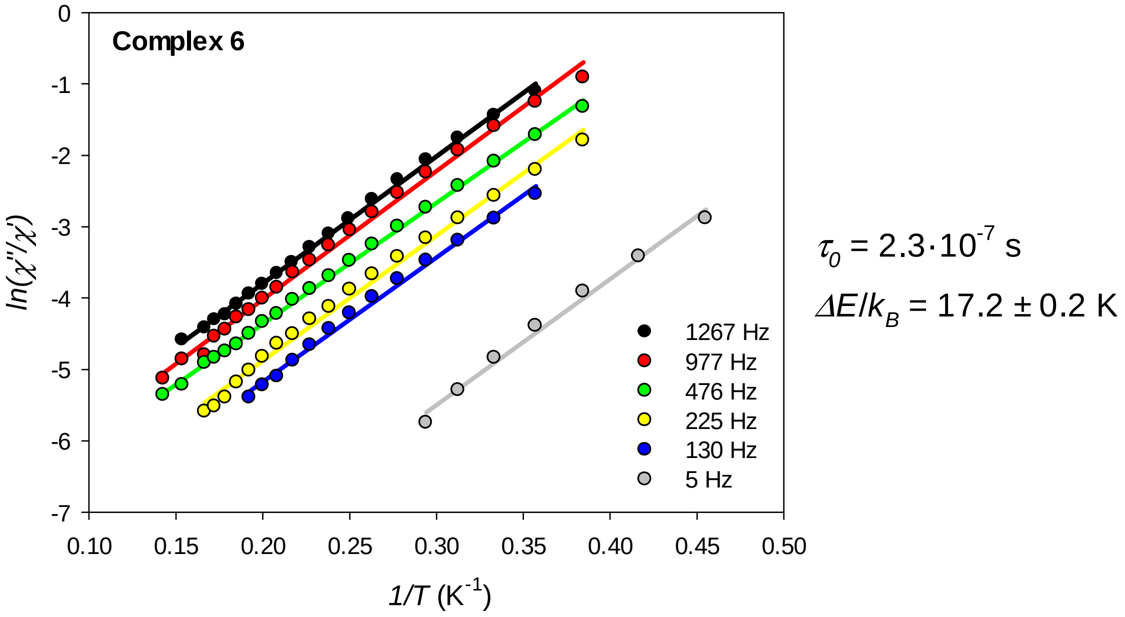

Fig S8 AC magnetic susceptibility of **7** in the absence of an external H_{DC} field (top), and natural logarithm of χ'/χ'' vs 1/T at selected frequencies of υ = 1267, 977, 476, 225, 130, 28, 10, 5, 1 Hz (bottom). Solid lines in χ'/χ'' vs 1/T correspond to fits of the data.[25]

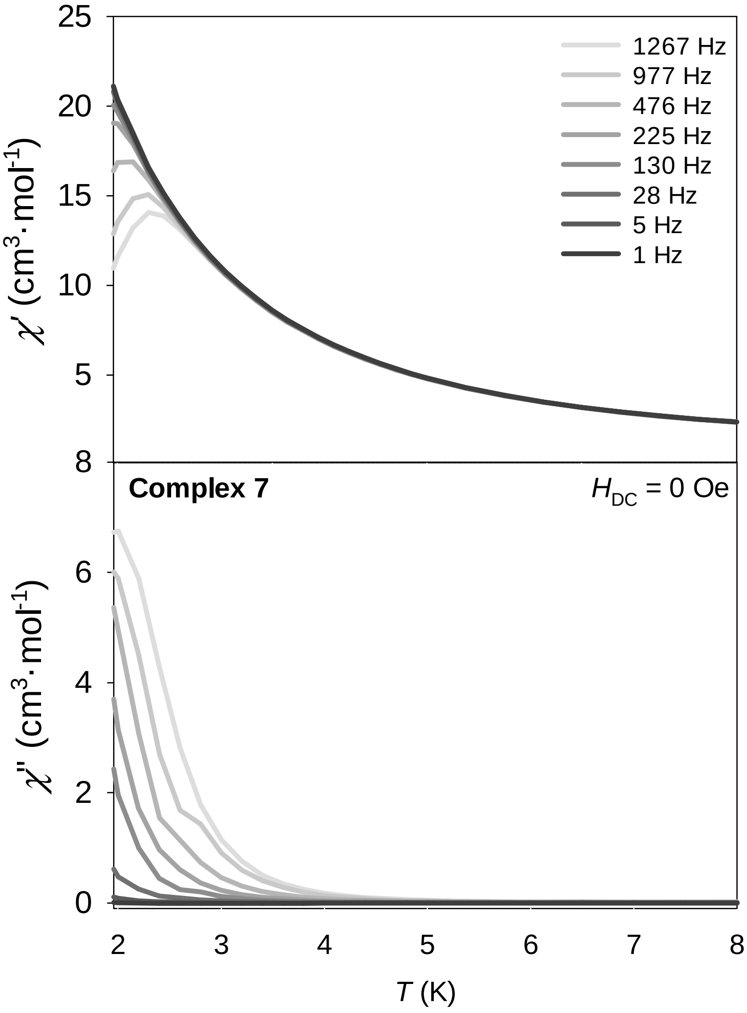

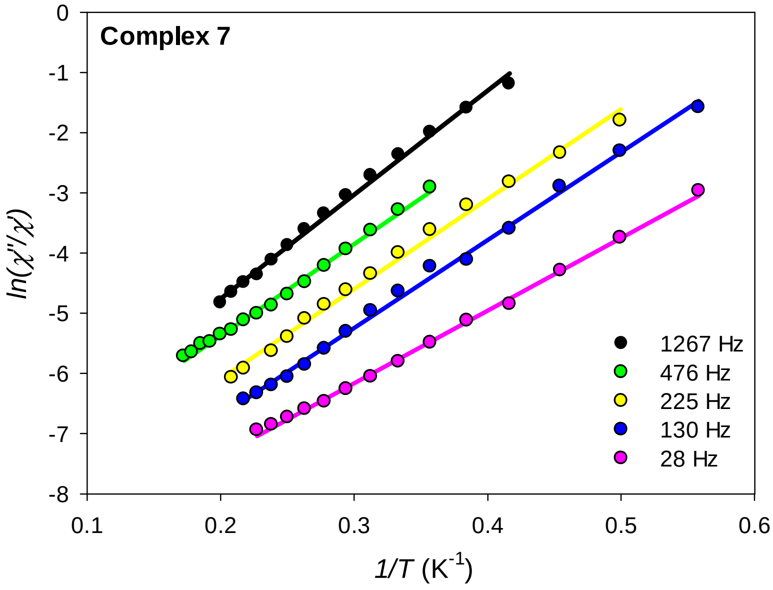

τ_0 = 1.2·10^{-7} s

$\Delta E/k_B$ = 14.8 ± 0.1 K

Table S7 Calculated energy spectrum, *g* tensors and angles (θ) of the principal anisotropy axes of excited states (ES) with respect to the ground state (GS) for model-1.

Level	Energy (cm^{-1})	g_z	Δ_{tun} (cm^{-1})	Angle (°)
1	0.00	17.79	0.45	-
2	0.45			
3	54.03	15.18	2.11	153.62
4	56.14			
5	96.95	12.33	7.14	137.79
6	104.09			
7	141.33	-	-	-
8	160.30	-	-	-
9	181.78	-	-	-
10	203.87	12.59	9.26	100.93
11	213.13			
12	309.73	17.35	0.92	100.58
13	310.65			

Table S8 Calculated energy spectrum, *g* tensors and angles (θ) of the principal anisotropy axes of excited states (ES) with respect to the ground state (GS) for model-2.

Level	Energy (cm^{-1})	g_z	Δ_{tun} (cm^{-1})	Angle (°)
1	0.00	17.80	0.08	-
2	0.08			
3	58.07	16.63	1.40	56.48
4	59.47			
5	115.66	12.13	3.73	24.12
6	119.39			
7	178.52	-	-	-
8	186.68	-	-	-
9	212.73	-	-	-
10	233.20	12.20	6.93	62.58
11	240.13			
12	295.48	17.15	3.41	94.42
13	298.89			

Table S9 Calculated energy spectrum, *g* tensors and angles (θ) of the principal anisotropy axes of excited states (ES) with respect to the ground state (GS) for complex model-3.

Level	Energy (cm^{-1})	g_z	Δ_{tun} (cm^{-1})	Angle (°)
1	0.00	17.72	0.32	-
2	0.32			
3	58.93	16.33	5.00	86.29
4	63.93			
5	98.94	-	-	-
6	125.34	-	-	-
7	134.39	-	-	-
8	184.73	11.81	5.05	77.73
9	189.78			
10	256.14	14.57	1.34	79.76
11	257.48			
12	371.81	17.59	0.75	92.65
13	372.56			

Figure S9 The structure of the calculated models (model-1, model-2 and model-3).

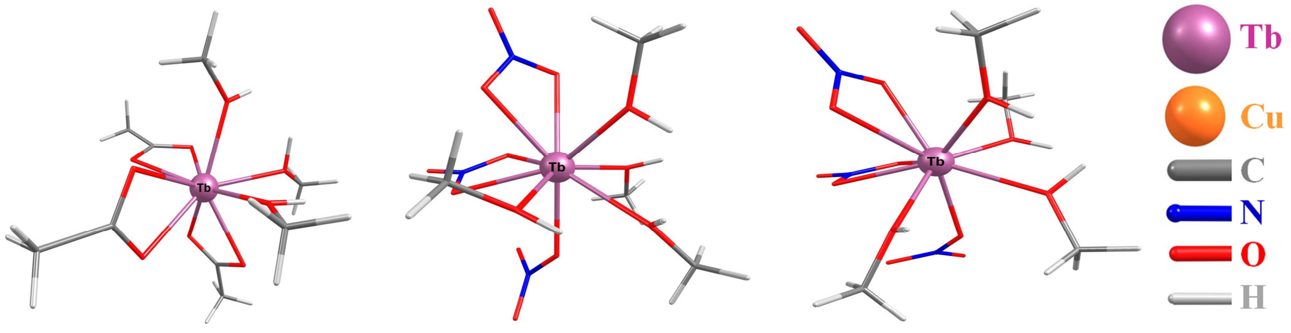

Model-1 (Complex-2) **Model-2 (Complex-4)** **Model-3 (Complex-4)**

Table S10 SINGLE_ANISO computed crystal field parameters for model-1, model-2 and model-3. The major components in the Table are in bold. B_k^q is the crystal field parameter and O_k^q is the extended Stevens operator. The quantization axis is chosen to be the main magnetic axis of the ground pseudo-doublet.

k	q	Model-1	Model-2	Model-3
		B_k^q	B_k^q	B_k^q
2	-2	-1.459	0.893	-3.752
	-1	**1.420**	**1.159**	**-0.912**
	0	**-2.016**	**-1.877**	**-1.996**
	1	**2.526**	**-2.667**	**-0.406**
	2	1.094	-1.672	-0.456
4	-4	-0.228×10^{-1}	-0.548×10^{-2}	0.184×10^{-1}
	-3	0.435×10^{-1}	-0.311×10^{-1}	0.104
	-2	0.167×10^{-1}	-0.822×10^{-2}	0.176×10^{-1}
	-1	-0.121×10^{-1}	-0.586×10^{-2}	0.619×10^{-2}
	0	-0.147×10^{-2}	-0.472×10^{-2}	-0.353×10^{-2}
	1	-0.293×10^{-1}	0.279×10^{-1}	0.430×10^{-3}
	2	0.163×10^{-2}	0.179×10^{-1}	-0.161×10^{-1}
	3	0.437×10^{-1}	-0.104	0.463×10^{-1}
	4	0.517×10^{-2}	-0.174×10^{-1}	0.299×10^{-2}
6	-6	0.218×10^{-3}	0.143×10^{-3}	0.222×10^{-3}
	-5	0.214×10^{-3}	0.523×10^{-3}	0.161×10^{-3}
	-4	0.887×10^{-5}	-0.190×10^{-4}	0.832×10^{-4}
	-3	0.255×10^{-3}	-0.862×10^{-4}	-0.175×10^{-3}
	-2	0.255×10^{-4}	0.137×10^{-3}	-0.415×10^{-4}
	-1	-0.131×10^{-3}	-0.291×10^{-3}	0.762×10^{-4}
	0	0.271×10^{-4}	0.581×10^{-6}	-0.342×10^{-4}
	1	0.151×10^{-3}	0.145×10^{-3}	0.803×10^{-5}
	2	0.109×10^{-3}	0.115×10^{-4}	0.139×10^{-3}
	3	-0.581×10^{-4}	0.328×10^{-3}	-0.224×10^{-3}
	4	0.109×10^{-3}	-0.669×10^{-4}	-0.120×10^{-3}
	5	0.764×10^{-3}	0.747×10^{-3}	0.948×10^{-3}
	6	-0.311×10^{-3}	0.204×10^{-4}	0.141×10^{-3}

References

[1] D. Casanova, M. Llunell, P. Alemany, S. Alvarez, *Chem. Eur. J.*, **2005**, *11*, 1479-1494.

[2] J.-P. Costes, M. Auchel, F. Dahan, V. Peyrou, S. Shova, W. Wernsdorfer, *Inorg. Chem.*, **2006**, *45*, 1924-1934.

[3] C. Aronica, G. Pilet, G. Chastanet, W. Wernsdorfer, J.-F. Jacquot, D. Luneau, *Angew. Chem. Int. Ed.*, **2006**, *45*, 4659-4662.

[4] J.-P. Costes, F. Dahan, W. Wernsdorfer, *Inorg. Chem.*, **2006**, *45*, 5-7.

[5] F. Mori, T. Nyui, T. Ishida, T. Nogami, K.-Y. Choi, H. Nojiri, *J. Am. Chem. Soc.*, **2006**, *128*, 1440-1441.

[6] T. Hamamatsu, K. Yabe, M. Towatari, N. Matsumoto, N. Re, A. Pochaba, J. Mrozinski, *Bull. Chem. Soc. Jpn.*, **2007**, *80*, 523-529.

[7] J.-P. Costes, S. Shova, W. Wernsdorfer, *Dalton Trans.*, **2008**, *14*, 1843-1849.

[8] T. Kajiwara, M. Nakano, S. Takaishi, M. Yamashita, *Inorg. Chem.*, **2008**, *47*, 8604-8606.

[9] T. Kajiwara, K. Takahashi, T. Hiraizumi, S. Takaishi, M. Yamashita, *Cryst. Eng. Comm*, **2009**, *11*, 2110-2116.

[10] T. Kajiwara, K. Takahashi, T. Hiraizumi, S. Takaishi, M. Yamashita, *Polyhedron*, **2009**, *28*, 1860-1863.

[11] V. Baskar, K. Gopal, M. Helliwell, F. Tuna, W. Wernsdorfer, R. E. P. Winpenny, *Dalton Trans.*, **2010**, *39*, 4747-4750.

[12] T. Kajiwara, M. Nakano, K. Takahashi, S. Takaishi, M. Yamashita, *Chem. Eur. J.*, **2011**, *17*, 196-205.

[13] S. K. Langley, L. Ungur, N. F. Chilton, B. Moubaraki, L. F. Chibotaru, K. S. Murray, *Chem. Eur. J.*, **2011**, *17*, 9209-9218.

[14] T. Shiga, H. Miyasaka, M. Yamashita, M. Morimoto, M. Irie, *Dalton Trans.*, **2011**, *40*, 2275-2282.

[15] Q. Zhu, S. Xiang, T. Sheng, D. Yuan, C. Shen, C. Tan, S. Hu and X. Wu, *Chem. Commun.*, **2012**, *48*, 10736-10738.

[16] T. Ishida, R. Watanabe, K. Fujiwara, A. Okazawa, N. Kojima, G. Tanaka, S. Yoshii, H. Nojiri, *Dalton Trans.*, **2012**, *41*, 13609-13619.

[17] J.-L. Liu, Y.-C. Chen, Q.-W. Li, S. Gomez-Coca, D. Aravena, E. Ruiz, W.-Q. Lin, J.-D. Leng, M.-L. Tong, *Chem. Commun.*, **2013**, *49*, 6549-6551.

[18] J.-L. Liu, W.-Q. Lin, Y.-C. Chen, S. Gómez-Coca, D. Aravena, E. Ruiz, J.-D. Leng, M.-L. Tong, *Chem. Eur. J.*, **2013**, *19*, 17567-17577.

[19] H. Zhang, G.-L. Zhuang, X.-J. Kong, Y.-P. Ren, L.-S. Long, R.-B. Huang and L.-S. Zheng, *Cryst. Growth Des.*, **2013**, *13*, 2493-2498.

[20] V. Chandrasekhar, A. Dey, S. Das, M. Rouzières, R. Clérac, *Inorg. Chem.*, **2013**, *52*, 2588-2598.

[21] X.-C. Huang, C. Zhou, H.-Y. Wei, X.-Y. Wang, *Inorg. Chem.*, **2013**, *52*, 7314-7316.

[22] I. A. Kuhne, N. Magnani, V. Mereacre, W. Wernsdorfer, C. E. Anson, A. K. Powell, *Chem. Commun.*, **2014**, *50*, 1882-1885.

[23] S. Ghosh, Y. Ida, T. Ishida, A. Ghosh, *Cryst. Growth Des.*, **2014**, *14*, 2588-2598.

[24] S. Xue, Y.-N. Guo, L. Zhao, H. Zhang, J. Tang, *Inorg. Chem.*, **2014**, *53*, 8165-8171.

[25] J. Bartolomé, G. Filoti, V. Kuncser, G. Schinteie, V. Mereacre, C. E. Anson, A. K. Powell, D. Prodius, C. Turta, *Phys. Rev. B*, **2009**, *80*, 014430.
